# An Albumin‐Photosensitizer Supramolecular Assembly with Type I ROS‐Induced Multifaceted Tumor Cell Deaths for Photodynamic Immunotherapy

**DOI:** 10.1002/advs.202410405

**Published:** 2025-01-13

**Authors:** Jingtian Zhang, Di Jiao, Xinwen Qi, Yufan Zhang, Xiaoang Liu, Tengwu Pan, Heqi Gao, Zhaoyun Liu, Dan Ding, Guangxue Feng

**Affiliations:** ^1^ Frontiers Science Center for Cell Responses State Key Laboratory of Medicinal Chemical Biology Key Laboratory of Bioactive Materials Ministry of Education and College of Life Sciences Nankai University Tianjin 300071 China; ^2^ Department of Hematology Tianjin Medical University General Hospital Tianjin Key Laboratory of Bone Marrow Failure and Malignant Hemopoietic Clone Control Tianjin Institute of Hematology Tianjin 300052 China; ^3^ Guangdong Provincial Key Laboratory of Luminescence from Molecular Aggregates State Key Laboratory of Luminescent Materials and Devices School of Materials Science and Engineering AIE Institute South China University of Technology Guangzhou 510640 China

**Keywords:** ferroptosis, immunogenic cell death, programmed cell death, pyroptosis, Type I photodynamic therapy

## Abstract

Photodynamic therapy holds great potentials in cancer treatment, yet its effectiveness in hypoxic solid tumor is limited by the oxygen‐dependence and insufficient oxidative potential of conventional type II reactive oxygen species (ROS). Herein, the study reports a supramolecular photosensitizer, BSA@TPE‐BT‐SCT NPs, through encapsulating aggregation‐enhanced emission photosensitizer by bovine serum albumin (BSA) to significantly enhance ROS, particularly less oxygen‐dependent type I ROS for photodynamic immunotherapy. The abundant type I ROS generated by BSA@TPE‐BT‐SCT NPs induce multiple forms of programmed cell death, including apoptosis, pyroptosis, and ferroptosis. These multifaceted cell deaths synergistically facilitate the release of damage‐associated molecular patterns and antitumor cytokines, thereby provoking robust antitumor immunity. Both in vitro and in vivo experiments confirmed that BSA@TPE‐BT‐SCT NPs elicited the immunogenic cell death, enhance dendritic cell maturation, activate T cell, and reduce myeloid‐derived suppressor cells, leading to the inhibition of both primary and distant tumors. Additionally, BSA@TPE‐BT‐SCP NPs also exhibited excellent antitumor performance in a humanized mice model, evidenced by a reduction in senescent T cells among these activated T cells. The findings advance the development of robust type I photosensitizers and unveil the important role of type I ROS in enhancing multifaceted tumor cell deaths and antitumor immunogenicity.

## Graphical abstract

Supramolecular photosensitizers BSA@TPE‐BT‐SCT NPs are developed through encapsulating aggregation‐enhanced emission photosensitizer by bovine serum albumin (BSA) to boost type I reactive oxygen species generation for photoimmunotherapy. BSA@TPE‐BT‐SCT NPs induce multifaceted cell deaths, including apoptosis, pyroptosis, and ferroptosis, and these deaths synergistically facilitate the release of damage‐associated molecular patterns and antitumor cytokines, thereby provoking robust antitumor immunity.

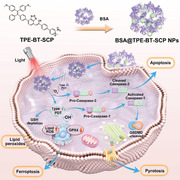

## 1 Introduction

Photodynamic therapy (PDT) has gained substantial research interest over recent decades, due to its non‐invasive nature, spatiotemporal selectivity, excellent biosafety, etc.^[^ ^1^, ^2^, ^3^, ^4^ ^]^ PDT relies on photosensitizers (PSs) to generate reactive oxygen species (ROS) upon localized light irradiation, causing oxidative stress in target cells that leads to cell death.^[^ ^5^ ^]^ Upon photon absorption, PSs in the ground singlet state (S_0_) are excited to the first excited singlet state (S_1_), reaching the triplet state (T_1_) through intersystem crossing (ISC), where they interact with substrates, usually oxygen (O_2_), to produce ROS.^[^ ^6^ ^]^ PSs are categorized into type I and type II groups based on the underlying photophysical and photochemical mechanisms. Type I PSs undergo electron or hydrogen transfer with substrates, resulting in the formation of free radicals such as superoxide anion (O_2_^·−^), hydroxyl radical (·OH), or hydrogen peroxide (H_2_O_2_) as type I ROS.^[^ ^7^ ^]^ Conversely, the type II PSs involve the energy transfer with O_2_, producing cytotoxic singlet oxygen (^1^O_2_).^[^ ^8^ ^]^ Currently, more than 20 PSs are either approved for clinical use or in clinical trials; however, most of these PSs are type II ones.^[^ ^9^ ^]^ Despite the prevalence, their antitumor efficacies are greatly limited in the hypoxic tumor microenvironment due to their high oxygen dependency.^[^ ^10^ ^]^

As type I PDT mainly involves the electron/hydrogen transfer, the less oxygen‐dependent nature has sparked the development of type I PSs.^[^ ^11^, ^12^, ^13^ ^]^ Great efforts have been made to enhance type I ROS production, such as, lowering T_1_ energy level, increasing intramolecular electron transfer ability, providing electron‐rich microenvironment, etc.^[^ ^14^, ^15^, ^16^, ^17^, ^18^ ^]^ Among them, supramolecular self‐assembly shows great promise.^[^ ^19^, ^20^, ^21^, ^22^ ^]^ For instance, Gao and colleagues used an electron‐rich polymer micelle to boost type I ROS generation of 5,10,15,20–tetrakis(meso‐hydroxyphenyl)porphyrin (mTHPP).^[^ ^23^ ^]^ Zheng and co‐authors demonstrated biotinylation could convert conventional type II PSs to type I.^[^ ^24^ ^]^ Yang et al. reported the supramolecular PSs through integration of BODIPY and perylene diimide for oxygen‐independent PDT.^[^ ^25^ ^]^ Despite these advances, most studies have mainly focused on the anti‐hypoxia prospect of type I PSs. While type I and type II ROS differ not only in their generation processes, but also in their reactivity with biological molecules. Type II ROS, i.e., ^1^O_2_, primarily reacts with electron‐rich targets such as carbon‐carbon double bonds, aromatic hydrocarbons, indoles, and so forth.^[^ ^26^ ^]^ In contrast, type I ROS, particularly ·OH, are highly potent oxidants capable of reacting with nearly all biological molecules, including lipids, DNA, RNA, proteins, and antioxidants.^[^ ^27^ ^]^ Notably, ·OH also facilitates the oxidization of monounsaturated fatty acids in cell membranes, a reaction that ^1^O_2_ cannot achieve, delivering new aspects in PDT.^[^ ^28^ ^]^ However, apart from hypoxia mitigation, the biological impacts of type I ROS remain less explored.

Recent discoveries of novel programmed cell death forms, such as ferroptosis and pyroptosis, offer new avenues for cancer immunotherapy.^[^ ^29^, ^30^, ^31^ ^]^ These programmed cell deaths are immunogenic, capable of inducing acute inflammatory responses and activating antitumor immunity.^[^ ^32^, ^33^, ^34^ ^]^ Ferroptosis mainly involves lipid peroxidation and glutathione peroxidase 4 (GPX4) inhibition, which releases immunogenic molecules that promote immune cell recruitment.^[^ ^35^, ^36^ ^]^ Pyroptosis is a highly inflammatory programmed cell death. It activates caspase‐1 and gasdermin D, releasing pro‐inflammatory cytokines that attract and activate immune cells.^[^ ^37^, ^38^ ^]^ Co‐activation of these pathways could great induce strong antitumor immunogenicity, and several inducers of pyroptosis and ferroptosis have been identified, but most of them involve metal species, raising concerns about potential toxicity and adverse effects.^[^ ^39^, ^40^ ^]^ Recent advancements, such as Tang and colleagues’ covalent organic framework (COF919), have shown promise in simultaneously activating both pathways in tumor cells.^[^ ^41^ ^]^ However, simpler, biocompatible nanomaterials and methods that regulate these cell death pathways remain limited and are highly desirable. Given the superior oxidative potential and broader reactivity spectrum of type I ROS, we hypothesize that developing robust type I PSs might provide a promising and attractive option for inducing multifaceted tumor cell deaths and boosting immunogenicity in PDT.

**Scheme 1:**
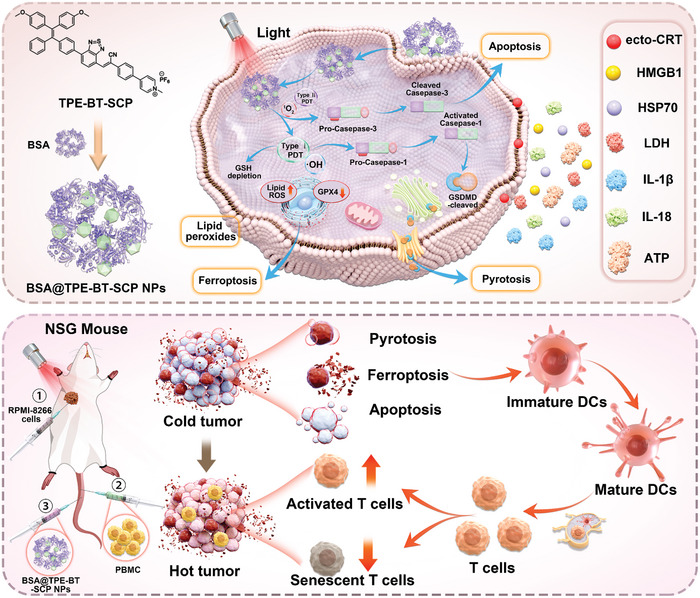
Schematic of the mechanism of BSA@TPE‐BT‐SCP NPs‐medicated multifaceted tumor cell deaths for inducing antitumor immunogenicity in PDT.

Herein, we present the design of a supramolecular PS, BSA@TPE‐BT‐SCP NPs, with superior type I ROS generation capability, and further demonstrate that the enhanced oxidative stress leads to multiple types of programmed cell death in tumors, significantly provoking robust antitumor immunity (**Scheme** **1**). The new PS, (Z)‐4‐(4‐(2‐(7‐(4‐(2,2‐bis(4‐methoxyphenyl)‐1‐phenylvinyl)phenyl)benzo[c][1,2,5]thiadiazol‐4‐yl)‐1‐cyanovinyl)phenyl)‐1‐methylpyridin‐1‐ium (TPE‐BT‐SCP) with aggregation‐enhanced emission (AIE) feature, was designed and further encapsulated by 1,2‐distearoyl‐sn‐glycero‐3‐phosphoethanolamine‐N‐[methoxy (polyethylene glycol)‐2000] (DSPE‐PEG_2000_) or bovine serum albumin (BSA) to afford PEG@TPE‐BT‐SCP NPs or BSA@TPE‐BT‐SCP NPs, respectively. Consistent with our previous findings,^[^ ^42^, ^43^ ^]^ BSA@TPE‐BT‐SCP NPs exhibited significantly better ROS generation ability over PEG@TPE‐BT‐SCP NPs, enhanced by ≈52 times under the same conditions. Notably, further investigation revealed that BSA@TPE‐BT‐SCP NPs exhibited potent type I ROS generation, and possessed more significant phototoxicity and superior anti‐tumor efficacy over PEG@TPE‐BT‐SCP NPs. Moreover, the enhanced oxidative stress from BSA@TPE‐BT‐SCP NPs led to multiple forms of programmed tumor cell death in tumors, including apoptosis, pyroptosis, and ferroptosis. The synergy of these multifaceted programmed cell deaths facilitated the effective release of damage‐associated molecular patterns (DAMPs), such as surface‐exposed calreticulin (eco‐CRT), heat shock protein 70 (HSP70), high mobility group protein 1 (HMGB1), among with antitumor cytokines, effectively provoking antitumor immunity (Scheme 1). Both in vitro and in vivo experimental results demonstrated that BSA@TPE‐BT‐SCP NPs induced dendritic cells (DCs) maturation and T cell activation, increased T cell infiltration, and reduced the proportion of immunosuppressive myeloid‐derived suppressor cells (MDSCs), thereby inhibiting both primary and distant tumors. Furthermore, BSA@TPE‐BT‐SCP NPs also exhibited excellent antitumor performance in a humanized mouse model, evidenced by increased activated T cells and decreased senescent T cells within the tumor microenvironment.

## 2 Results and Discussion

**Figure 1:**
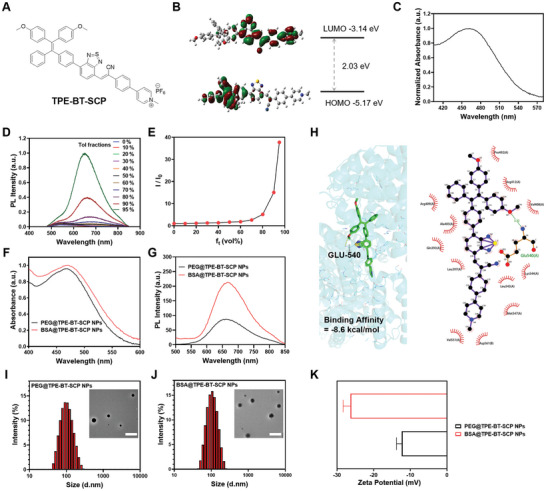
Synthesis and Characterization of PSs. A) Chemical structure and B) HOMO‐LUMO distribution of TPE‐BT‐SCP. C) Absorption spectrum of TPE‐BT‐SCP in DMSO solution. D) PL spectra of TPE‐BT‐SCP in DMSO/Tol mixture with varying Tol fractions. E) Relative PL intensity changes of TPE‐BT‐SCP versus *f_t_*. F) Absorption and G) PL spectra of PEG@TPE‐BT‐SCT NPs and BSA@TPE‐BT‐SCT NPs, respectively. H) Detailed bindings between TPE‐BT‐SCP and BSA proposed from molecular docking studies. Hydrodynamic size distributions of I) PEG@TPE‐BT‐SCT NPs and (J) BSA@TPE‐BT‐SCT NPs, insets are the corresponding TEM images (Scale bar = 500 nm. K) Zeta potentials of PEG@TPE‐BT‐SCT NPs and BSA@TPE‐BT‐SCT NPs. Data presented as mean ± SD, n = 3.

A donor‐acceptor type PS with AIE characteristics (**Figure** **1**A), named TPE‐BT‐SCP, was synthesized. The classic AIE unit, methoxy‐substituted tetraphenylethylene (TPE), served as the electron donor, while benzothiadiazole (BT) and electron‐deficient cationic pyridine acted as the π‐bridge and electron acceptor, respectively. Notably, the cationic pyridine unit facilitates radical‐type ROS generation.^[^ ^15^ ^]^ The synthetic route is shown in Figure S1 (Supporting Information), and the chemical structures were confirmed by ^1^H and ^13^C NMR and high‐resolution mass spectrometry (HR‐MS) (Figures S2–S7, Supporting Information). Density functional theory (DFT) calculation provided structural insights into TPE‐BT‐SCP. As shown in Figure 1B, the geometry at the S_0_ energy level indicated that the highest occupied molecular orbital (HOMO) is localized on the electron donor TPE segment, while the lowest unoccupied molecular orbital (LUMO) is mainly distributed on the cationic pyridine acceptor. The spatial separation of HOMO and LUMO is conducive for ISC process and triplet state generation, and hence the production of ROS. The HOMO/LUMO energy levels of TPE‐BT‐SCP are ‐5.17/‐3.14 eV, respectively. The UV–vis absorption spectrum revealed a maximum absorption wavelength of 465 nm for TPE‐BT‐SCP in dimethyl sulfoxide (DMSO) (Figure 1C). To further verify the AIE feature of TPE‐BT‐SCP, the photoluminescence (PL) spectra were measured at different DMSO/Toluene (Tol) ratios (Figure 1D,E). In pure DMSO, TPE‐BT‐SCP exhibited an emission peak at 580 nm, which red‐shifted to 645 nm in the aggregate state (DMSO/Tol = 5:95) (Figure 1D), due to the intramolecular charge transfer feature of the D‐A configuration. With the addition of Tol as the poor solvent to DMSO to enable aggregate formation, the PL intensities of TPE‐BT‐SCP significantly intensified, with an enhancement factor of ≈38 at the Tol faction (*f_t_*) of 95%, proving the potent AIE feature of TPE‐BT‐SCP.

To enhance the colloidal stability and biocompatibility, TPE‐BT‐SCP was encapsulated into two distinct types of organic nanoparticles using a modified nanoprecipitation technique.^[^ ^42^ ^]^ DSPE‐PEG_2000_ (an amphiphilic polymer) and BSA (nature protein) were used as encapsulation matrices, forming PEG@TPE‐BT‐SCP NPs and BSA@TPE‐BT‐SCP NPs, respectively. The loading efficiencies of TPE‐BT‐SCP in BSA@TPE‐BT‐SCP NPs and PEG@TPE‐BT‐SCP NPs were determined to be ≈20% and ≈1%. BSA@TPE‐BT‐SCP NPs exhibited higher absorbance than PEG@TPE‐BT‐SCP NPs (Figure 1F), attributed to more restricted intramolecular motions and planarized molecular geometry within the intraparticle microenvironment of BSA pocket. The emission peaks of PEG@TPE‐BT‐SCP NPs and BSA@TPE‐BT‐SCP NPs were located at 660 nm and 680 nm, respectively, with BSA@TPE‐BT‐SCP NPs showing stronger fluorescence emission intensity due to hydrophobic van der Waals interactions, further restricting molecular motion and enhancing brightness (Figure 1G). Docking and molecular modeling studies confirmed the tight binding interactions and binding sites between BSA and TPE‐BT‐SCP (Figure 1H).^[^ ^17^, ^44^ ^]^ TPE‐BT‐SCP formed hydrogen bonds with over 10 amino acid residues of BSA pockets, with a binding energy of ‐8.6 kcal/mol, further indicating the strong interaction between BSA and TPE‐BT‐SCP.^[^ ^45^ ^]^ Dynamic light scattering (DLS) analysis revealed the average hydrodynamic diameters of ≈100 and ≈109 nm for PEG@TPE‐BT‐SCP NPs and BSA@TPE‐BT‐SCP NPs, respectively. Transmission electron microscopy (TEM) images suggested the similar and uniform spherical morphologies for both nanoparticles with sizes ≈100 nm (Figure 1I,J), in alignment with the DLS results. In addition, the surface zeta potential were ‐12.3 mW for PEG@TPE‐BT‐SCP NPs and ‐26.3 mV for BSA@TPE‐BT‐SCP NPs, suggesting the presence of PEG and BSA at nanoparticle surface and the successful TPE‐BT‐SCP encapsulation. Both nanoparticles also exhibited excellent photostability, as manifested by the minimal absorbance changes under continuous light irradiation (Figure S8, Supporting Information). In addition, their sizes remain nearly unchanged after being stored in fetal bovine serum (FBS) solution or in acetate buffer solutions with pH 5 for 7 days (Figure S9, Supporting Information), suggesting their excellent colloidal stability in the cellular lysosomal microenvironment and the high potential for in vivo biological applications.

**Figure 2:**
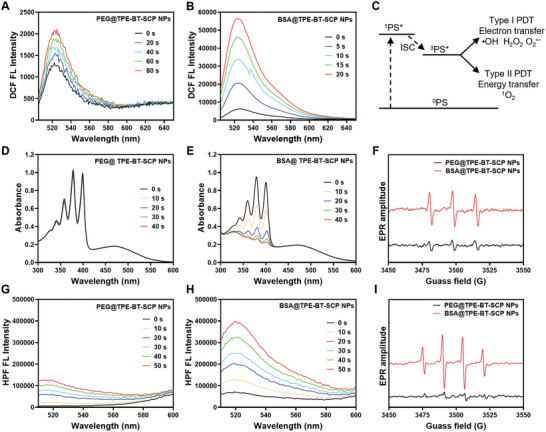
ROS generation analysis. A,B) PL spectra changes of DCFH in the presence of (A) PEG@TPE‐BT‐SCP NPs and (B) BSA@TPE‐BT‐SCP NPs, under white light irradiation (0.2 W cm^−2^, NPs concentration: 5 µm). C) Schematic of ROS generation mechanism. D,E) Absorption spectra changes of ABDA in the presence of (D) PEG@TPE‐BT‐SCP NPs and (E) BSA@TPE‐BT‐SCP NPs (5 µm) upon white light irradiation (0.2 W cm^−2^). F) EPR analysis with TEMP as the trapping agent. G,H) PL spectra changes of HPF in the presence of (G) PEG@TPE‐BT‐SCP NPs (5 µm) and (H) BSA@TPE‐BT‐SCP NPs (5 µm), under white light irradiation (0.2 W cm^−2^, 30s). I) EPR analysis with DMPO as the trapping agent.

To access the ROS generation performance of PEG@TPE‐BT‐SCP NPs and BSA@TPE‐BT‐SCP NPs, 2`,7`‐dichlorodihydrofluorescein (DCFH) as an overall ROS indicator was used, which shows intensified green fluorescence upon reaction with all types of ROS.^[^ ^46^, ^47^ ^]^ As shown **Figure** **2**A, PEG@TPE‐BT‐SCP NPs exhibited a moderate DCFH fluorescence light‐up ability, with a fluorescence enhancement factor of 1.65 after 80 sec of white light irradiation. In contrast, BSA@TPE‐BT‐SCP NPs showed a dramatic 52‐fold increase in ROS generation under identical conditions (Figure 2A,B; Figure S10, Supporting Information), proving that BSA loading is a more effective approach to develop supramolecular PSs. To further explore the mechanism underlying the enhanced ROS generation by BSA@TPE‐BT‐SCP NPs, we first investigated the ROS generation capacity of TPE‐BT‐SCP molecules in different aggregate states. As shown in Figure S11 (Supporting Information), TPE‐BT‐SCP showed minimal ROS generation in pure DMSO, while addition of Tol to induce aggregate formation greatly promoted ROS generation, confirming the aggregation‐enhanced ROS generation effect of TPE‐BT‐SCP.

ROS generated by PDT can be classified into type I ROS and type II ROS based on their photophysical and photochemical properties.^[^ ^48^ ^]^ The types of generated ROS were then identified using specific ROS probes. 9,10‐Anthracenediyl‐bis (methylene) dimalonic acid (ABDA) was first used to explore the type II ROS (^1^O_2_) production ability.^[^ ^49^ ^]^ PEG@TPE‐BT‐SCP NPs resulted in a relatively slow ABDA decomposition rate, while BSA@TPE‐BT‐SCP NPs could readily decompose all ABDA within 40 sec of irradiation, demonstrating 20‐fold higher ¹O₂ production compared to PEG@TPE‐BT‐SCP NPs (Figure 2D,E; Figure S12, Supporting Information). Electron paramagnetic resonance (EPR) analysis with TEMP as the ^1^O_2_ trapping agents also demonstrated stronger characteristic peaks of TEMP‐^1^O_2_ for BSA@TPE‐BT‐SCP NPs,^[^ ^50^ ^]^ further supporting their pronounced ^1^O_2_ generation ability over PEG@TPE‐BT‐SCP NPs (Figure 2F). The type I ROS generation ability was subsequently verified with 4‐hydroxyphenyl‐fluorescein (HPF) as the ·OH probe,^[^ ^51^ ^]^ which reacts with ·OH to yield intense green fluorescence centered at 520 nm. Under the same white light irradiation (0.2 W cm^−2^), BSA@TPE‐BT‐SCP NPs showed a 2.46‐fold higher fluorescence enhancement than PEG@TPE‐BT‐SCP NPs under identical conditions (Figure 2G,H; Figure S13, Supporting Information). 5,5‐Dimethyl‐1‐pyrroline N‐oxide (DMPO) was further used as a ·OH and O_2_^·−^ trapping agent for EPR analysis.^[^ ^52^ ^]^ Intriguingly, only the characteristic peaks of DMPO‐·OH were observed and BSA@TPE‐BT‐SCP NPs yielded much stronger DMPO‐·OH peaks over PEG@TPE‐BT‐SCP NPs (Figure 2I), suggesting the excellent and predominate ·OH generation capability of BSA@TPE‐BT‐SCP NPs. Besides, the O_2_^·−^specific fluorescence probe dihydrorhodamine 123 (DHR123) was also used to evaluate the O_2_^·−^ production capacity,^[^ ^53^ ^]^ which revealed that they hardly produced O_2_^·−^ (Figure S14, Supporting Information), in consistence with the conclusion of EPR analysis. BSA@TPE‐BT‐SCP NPs also maintained very strong ROS generation ability under hypoxic condition (Figure S15, Supporting Information), due to the less‐oxygen dependent nature of type I ROS generation pathway. Therefore, our findings manifest that the ROS generation capability of PSs could be further modulated and enhanced by using different carriers, whereas BSA as the loading cargo demonstrated excellent both ^1^O_2_ and ·OH generation capabilities over the widely adopted lipid‐PEG matrices. Femtosecond transient absorption and cyclic voltammetry (CV) measurements were further conducted to investigate the mechanism of BSA‐enhanced type I ROS generation. Femtosecond transient absorption study showed reduced ground‐state bleaching band at 500 nm and excited‐state absorption (ESA) band between 600–750 nm for BSA@TPE‐BT‐SCP NPs as compared to pure TPE‐BT‐SCP molecules in argon atmosphere, suggesting the occurrence of electron transfer from BSA to TPE‐BT‐SCP (Figure S16, Supporting Information).^[^ ^17^ ^]^ Further CV measurement indicated gradually reduced reduction currents with successive electrochemical scanning cycles, suggesting the formation of electrochemically nonactive complex between BSA and photosensitizer as a result of their electron transfer. CV measurement also proved the thermodynamically favored electron transfer from PS^·−^ to O_2_ to form O_2_^·−^.^[^ ^54^ ^]^ In addition, the ESA lifetimes of TPE‐BT‐SCP and BSA@TPE‐BT‐SCP NPs were further reduced in oxygen atmosphere (Figure S16, Supporting Information), suggesting the potential electron transfer from these PSs to surrounding oxygen.

**Figure 3:**
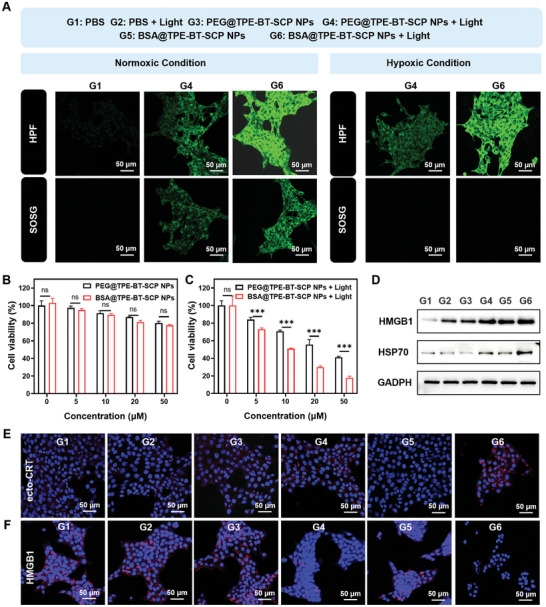
In vitro ROS generation and ICD induction. A) CLSM images of intracellular ROS generation from different groups. B) Cell viabilities of 4T1 cells incubated with PEG@TPE‐BT‐SCP NPs or BSA@TPE‐BT‐SCP NPs without while light irradiation. C) Cell viabilities of 4T1 cells incubated with PEG@TPE‐BT‐SCP NPs or BSA@TPE‐BT‐SCP NPs followed by while light irradiation (0.2 W cm^−3^, 2 min). D) WB analysis of the expression of HMGB1 and HSP70 from 4T1 cells after different treatments. E) CLSM images of ecto‐CRT expression from 4T1 cells after different treatments. (F) CLSM images of HMGB1 expression from 4T1 cells after different treatment. Data presented as mean ± SD, n = 4. p* < 0.05, p** < 0.01, p*** < 0.001.

The antitumor effect of BSA@TPE‐BT‐SCP NPs was evaluated firstly in vitro, using 4T1 breast cancer cells. The cellular internalization of PEG@TPE‐BT‐SCP NPs and BSA@TPE‐BT‐SCP NPs into 4T1 cells was studied by qualitative confocal laser scanning microscopy (CLSM). CLSM images revealed bright fluorescence from TPE‐BT‐SCP inside 4T1 cells, indicating their effective cellular uptake. In addition, the intracellular fluorescence of both nanoparticles all overlapped very well with the fluorescence from lysosome tracker (Figure S17, Supporting Information), indicating an endocytosis internalization pathway. Furthermore, two cell‐permeable fluorogenic ROS detection probes, HPF and singlet oxygen‐sensor‐green (SOSG), were used to measure the intracellular ·OH and ^1^O_2_ production of these nanoparticles in 4T1 cells under both normoxic condition (21% O_2_) and hypoxic conditions (8% O_2_).^[^ ^47^, ^55^ ^]^ 4T1 cells were sequentially incubated with PEG@TPE‐BT‐SCP NPs or BSA@TPE‐BT‐SCP NPs for 6 h, HPF or SOSG for 30 min. Subsequently, these cells were irradiated with white light and subjected to CLSM imaging (**Figure** **3**A). Under normoxic conditions, HPF showed weak intracellular green fluorescence in PEG@TPE‐BT‐SCP NPs + Light group (G4) while intense green fluorescence in BSA@TPE‐BT‐SCP NPs + Light group (G6), indicating that BSA@TPE‐BT‐SCP NPs generated much more abundant ·OH in 4T1 cells. The intracellular fluorescence signal of SOSG also exhibited a similar trend, consistent with our results in solution testing. However, under hypoxic conditions, egligible intracellular fluorescence from SOSG was observed across all groups, while HPF still emitted strongly in BSA@TPE‐BT‐SCP NPs + Light group (G6). The sharp contrast clearly proves that BSA@TPE‐BT‐SCP NPs robustly generated ·OH even under hypoxic condition. Further semiquantitative analysis of intracellular HPF intensities confirmed strong ·OH generation under normoxic and hypoxic conditions, highlighting the less oxygen‐dependent Type I ROS generation by BSA@TPE‐BT‐SCP NPs (Figure S18, Supporting Information). Subsequently, the viability of 4T1 cells was accessed. The results indicated that both BSA@TPE‐BT‐SCP NPs and PEG@TPE‐BT‐SCP NPs exhibited minimal dark toxicity without light exposure (Figure 3B). Nevertheless, light treatment on cells pre‐incubated with these nanoparticles leads to significant cell death in dose‐dependent manners. Notably, BSA@TPE‐BT‐SCP NPs, with more ROS especially ·OH generation ability, exhibited a more pronounced tumor‐killing effect with a half‐maximum inhibition concentration (*IC*_50_) of 10.3 µm, compared to 24.9 µM for PEG@TPE‐BT‐SCP NPs (Figure 3C). Live/dead staining also suggested the better cell killing effect of BSA@TPE‐BT‐SCP NPs (Figure S19, Supporting Information), corroborating to the cell viabi results.

Previous researches, including our own, have confirmed that the ROS generated by PDT can induce immunogenic cell death (ICD), and thereby triggering antitumor immunity.^[^ ^42^, ^56^ ^]^ However, most studies predominately focused on type II PSs that primarily generated ^1^O_2_. We further examined whether the excellent type I ROS generation capability of BSA@TPE‐BT‐SCP NPs could effectively induce ICD in 4T1 cells. ICD process is characterized by the translocation of calreticulin (ecto‐CRT) from the endoplasmic reticulum to the cell membrane surface and the release of DAMPs such as HMGB1 and HSP70.^[^ ^57^, ^58^, ^59^ ^]^ The release of HMGB1 and HSP70 were evaluated by western blot (WB) experiments. As shown in Figure 3D, 4T1 cells treated with PEG@TPE‐BT‐SCP NPs and light irradiation (G4) or BSA@TPE‐BT‐SCP NPs and light irradiation (G6) exhibited elevated levels of HMGB1 and HSP70 in comparison with the PBS control group (G1). G6 exhibited notably higher HSP70 level over G4, clearly suggesting the better DAMPs release capability of BSA@TPE‐BT‐SCP NPs in PDT treatment. CLSM images and semiquantitative analysis further indicated the substantial expression of ecto‐CRT (Figure 3E; Figure S20, Supporting Information) and the marked decrease of intracellular HMGB1 (Figure 3F; Figure S21, Supporting Information) in G6 compared to G4 and all other groups. Collectively, these results together demonstrated that BSA@TPE‐BT‐SCP NPs could induce a much more robust ICD effect in tumor cells, which should be due to their abundant ROS production ability under light irradiation.

**Figure 4:**
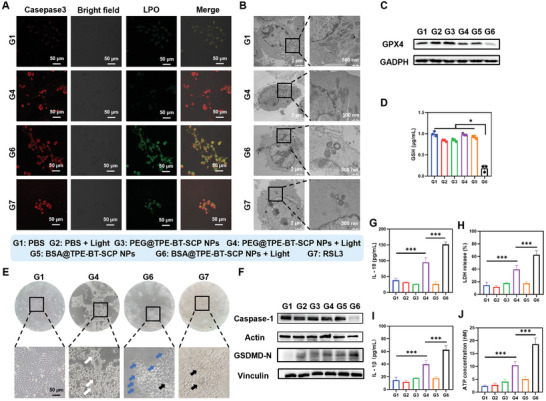
Multifaceted cell deaths induced by BSA@TPE‐BT‐SCP NPs. A) CLSM images of casepase‐3 and LPO expression from 4T1 cells in different groups. B) TEM images to show mitochondria damage in 4T1 cells after different treatments. C) WB analysis of GPX4 expression in 4T1 cells from different groups. D) GSH levels inside 4T1 cells after different treatments. E) Microscope images of 4T1 cells from different groups. F) WB analysis of caspase‐1 and GSDMD‐N expression in 4T1 cells from different groups. G–J) The levels of (G) IL‐18, (H) LDH, (I) IL‐1β, and (J) ATP in 4T1 cells from different groups. Data presented as mean ± SD, n = 3. p* < 0.05, p** < 0.01, p*** < 0.001.

An imbalance in cellular redox can cause oxidative damage to cellular components. ^1^O_2_ reacts with electron‐rich molecules, such as indoles, carbon‐carbon double bonds, aromatic hydrocarbons, and heterocyclic aromatics., etc., whereas ·OH is much more reative, capable of reacting with nearly all biological molecules, including lipids, RNA, DNA, and proteins.^[^ ^27^ ^]^ Specially, ·OH can oxidize polyunsaturated fatty acid residues on cellular membrane while ^1^O_2_ cannot.^[^ ^28^ ^]^ Given the fact that ferroptosis often results from lipid peroxidation of cell membrane,^[^ ^35^, ^60^, ^61^ ^]^ we hypothesize that BSA@TPE‐BT‐SCP NPs may profoundly induce ferroptosis. To verify our hypothesis, we assessed the levels of lipid peroxide (LPO) and caspase‐3 protein within the cells, which serve as markers for ferroptosis and apoptosis, respectively, using a lipid peroxidation sensor BODIPY581/591‐C11 and a caspase‐3 antibody^[^ ^62^, ^63^ ^]^. CLSM images and semiquantitative analysis revealed green fluorescence in the BSA@TPE‐BT‐SCP NPs + Light group and RSL3 (a ferroptosis inducer) group, while negligible green emission in PEG@TPE‐BT‐SCP + Light group (**Figure** **4**A; Figure S22, Supporting Information), indicating high lipid peroxide (LPO) levels could only be caused by the abundant intracellular ROS.^[^ ^64^ ^]^ On the other hand, the red fluorescence signal, indicative of caspase‐3 activation, emerged in PEG@TPE‐BT‐SCP NPs + Light group, BSA@TPE‐BT‐SCP NPs + Light group, and RSL3 group, confirming the occurrence of apoptosis (Figure 4A; Figure S23, Supporting Information). These results suggest that apoptosis occurred in PEG@TPE‐BT‐SCP NPs + Light group, while both apoptosis and ferroptosis occurred in BSA@TPE‐BT‐SCP NPs + Light group. Additionally, it has been reported that cell mitochondria exhibited shrinkage when cells undergo ferroptosis,^[^ ^65^ ^]^ TEM images indeed showed shrunken mitochondria morphologies in BSA@TPE‐BT‐SCP NPs + Light (G6) and RSL3 (G7) groups (Figure 4B). Moreover, the GPX4 protein expression (Figure 4C) and GSH levels (Figure 4D) were also significantly downgraded, consistent with the LPO results, further validating that both apoptosis and ferroptosis occurred in BSA@TPE‐BT‐SCP NPs + Light group.

The substantial generation of intracellular ROS during PDT can also lead to acute inflammation and induce pyroptosis, a form of ICD. Pyroptosis is driven by caspase‐induced cleavage of gasdermin (GSDM), resulting in pore formation on cell membrane and the release of IL‐1β, lactate dehydrogenase (LDH), eventually leading to cell lysis.^[^ ^66^ ^]^ It has been previously reported that activation of either caspase‐1 or caspase‐3 pathway can cleave GSDM and trigger pyroptosis.^[^ ^66^ ^]^ The potential of BSA@TPE‐BT‐SCP in inducing pyroptosis was first evaluated by monitoring cellular morphology and membrane changes after light irradiation. Interestingly, significant pyroptosis‐bubbles were observed on the membranes of 4T1 cells in BSA@TPE‐BT‐SCP NPs + Light group (G6) under the optical microscope (Figure 4E), suggesting these cells were undergoing pyroptosis as a third form of cell death in addition to apoptosis and ferroptosis. WB results further confirmed the occurrence of pyroptosis in BSA@TPE‐BT‐SCP NPs + Light group, as manifested the activation of casepase‐1 and the cleavage of GSDMD from WB observation (Figure 4F). In addition, other pyroptosis makers in these groups were also analyzed. As shown in Figure 4G–J, only PSs and light irradiation (G4 and G6 groups) could readily upregulate the levels of IL‐18, IL‐1β, LDH and ATP, whereas G6 group presented the highest levels among all groups. These results confirmed that 4T1 tumor cells treated with BSA@TPE‐BT‐SCP underwent pyroptosis, apoptosis and ferroptosis, and the synergy of these multifaceted tumor death pathways could remarkably induce a robust ICD effect, thus significantly promoting the antitumor immunity.

Given the excellent antitumor and immune activation effects of BSA@TPE‐BT‐SCP NPs observed in vitro, we moved to evaluate their effectiveness in vivo. The biosafety was initially accessed. Hemolysis assay suggested that these nanoparticles do not adversely affect red blood cells (Figure S24, Supporting Information), indicating their excellent biocompatibility. After four consecutive doses (1 mg/mL, 200 µL, on day 0, 2, 4, 6), the mice were sacrificed on day 9, their main organs and blood were collected. Hematoxylin and eosin (H&E) staining of main organs from mice treated with PEG@TPE‐BT‐SCP NPs and BSA@TPE‐BT‐SCP NPs showed no pathological changes compared to control group (Figure S25, Supporting Information), indicating minimal in vivo toxicity. Besides, blood biochemical analysis showed no significant difference between healthy mice and the mice injected with both nanoparticles in terms of hepatic and renal functions (Figure S26, Supporting Information). The biodistribution of these nanoparticles was then monitored after intravenous injection of PEG@TPE‐BT‐SCP NPs or BSA@TPE‐BT‐SCP NPs. As shown in Figure S27 (Supporting Information), the tumor site fluorescence, consecutively monitored at different time points (0, 2, 4, 8, 12, and 24 h), suggested that the accumulation of both PEG@TPE‐BT‐SCP NPs and BSA@TPE‐BT‐SCP NPs in the tumor tissue increased over time. BSA@TPE‐BT‐SCP NPs started to accumulate at tumor site at 0.5 h post injection, and the retention could last over 24 h. In contrast, PEG@TPE‐BT‐SCP NPs showed a slower tumor accumulation (at 2 h post injection) and faster tumor clearance (within 24 h post injection). The large difference is likely due to the superior long‐circulating capability of BSA‐based nanoparticles, thus enhancing tumor accumulation over time.^[^ ^67^, ^68^ ^]^ Ex vivo fluorescence images of organs collected at 8 h post intravenous injection showed that both nanoparticles could accumulate in tumors, while BSA@TPE‐BT‐SCP NPs showed a much better tumor accumulation ability (Figure S28, Supporting Information).^[^ ^67^ ^]^

**Figure 5:**
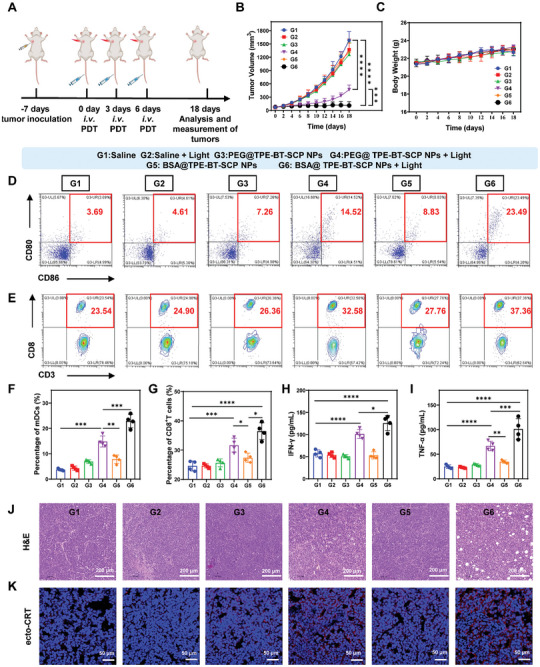
In vivo inhibition of 4T1 tumor by BSA@TPE‐BT‐SCP NPs. A) Schematic of in vivo PDT treatment of 4T1‐bearing mice. B) Tumor growth curves of mice from different groups. C) Body weight growth curves of mice from different groups. D) Flow cytometry analysis of mature DCs (mDCs) (CD80^+^ CD86^+^, gated on CD11c^+^) in draining lymph nodes. E) Flow cytometry analysis of CD8^+^CD3^+^ T cells in the tumor tissues from different groups. Percentages of F) mature DCs, G) CD8^+^CD3^+^ T cells, and secretion levels H) IFN‐γ and I) TNF‐α in tumor tissues from different groups. J) H&E staining images of tumor tissues from different groups. K) Immunofluorescence staining images of ecto‐CRT expression in tumor tissues from different groups. Data presented as mean ± SD, n = 4. p* < 0.05, p** < 0.01, p*** < 0.001, p**** < 0.0001.

To evaluate the PDT effect in vivo, we established a 4T1 tumor‐bearing mouse model (**Figure** **5**A). Once the tumor volume reached to 75 mm^3^, the mice were randomly assigned to six groups (n = 4 mice per group): Saline (G1); Saline + Light (G2); PEG@TPE‐BT‐SCP NPs (G3); PEG@TPE‐BT‐SCP NPs + Light (G4); BSA@TPE‐BT‐SCP NPs (G5); BSA@TPE‐BT‐SCP NPs + Light (G6). The mice in G3, G4, G5, G6 received NPs intravenously on days 0, 3, and 6, with those in G4 and G6 receiving white light irradiation (0.2 W cm^−2^, 8 min) at 8 h after injection. The tumor growth was consecutively monitored for 18 days. As shown in Figure 5B, the tumor growth was effectively inhibited in G4 and G6 groups, where the tumor volume in G4 group on day 18 was only 0.25‐fold of that in G1 group. Tumor growth was nearly completely inhibited in G6 group, attributed to the much more excellent ROS generation ability by BSA@TPE‐BT‐SCP NPs. Besides, body weight changes in all groups shown no significant difference (Figure 5C), further indicating their excellent biosafety.

To confirm the immune activation effects of PDT in vivo, immunofluorescence staining with flow cytometry was employed to analyze immune cells in tumors and draining lymph nodes of tumor‐bearing mice. Previous reports have shown that tumor cells undergoing ICD can activate DCs, which in turn present antigens to CD8^+^ T cells, thereby activating adaptive antitumor immunity. Therefore, the DCs maturation in nearby lymph nodes was first evaluated. As shown in Figure 5D,F, both G4 and G6 groups exhibited upregulated expressions of CD86 and CD80, the biomarkers of mature DCs (mDCs), whereas G6 group show much higher levels compared to G4 group, suggesting the ICD induction could readily mediate DC maturation while abundant generation of more oxidative type I ROS is more prone to DC maturation, as these powerful ROS could induce multiple types of ICD in the tumor cells. In addition, the intratumoral levels of CD8^+^ T cells also drastically increased in G4 and G6 groups compared to other groups, reaching 32% and 38%, respectively, proving the activation of cytotoxic T cells (Figure 5E,G). Additionally, the enzyme‐linked immunosorbent assay (ELISA) measurement of IFN‐γ (Figure 5H) and TNF‐α (Figure 5I) levels in the tumor supernatant also showed the highest cytokine secretion in G6 group, proving the systemic immune response activation. H&E staining of tumors revealed evident tumor cell death in G6 group (Figure 5J), and immunofluorescence staining also suggested much more significant ecto‐CRT expression on the tumor cells in G6 group (Figure 5K), confirming the best ICD induction brought by BSA@TPE‐BT‐SCP NPs under light irradiation. We and other have demonstrated that the generated ROS could elicit ICD in tumor cells, however, the ICD induction mainly involved one specific programmed cell death pathway.^[^ ^32^ ^]^ In this work, the tremendous type I ROS generation from BSA@TPE‐BT‐SCP NPs leads to multifaced cell deaths, including apoptosis, pyroptosis and ferroptosis, which cooperatively strengthens T cell‐mediated antitumor immune responses and effectively inhibits tumor growth.

**Figure 6:**
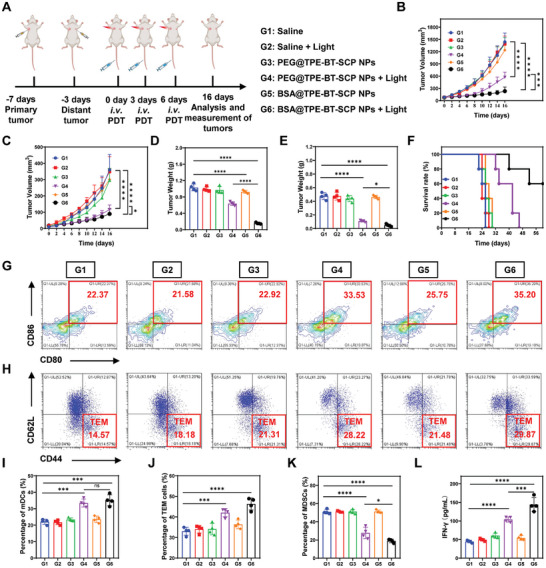
Long‐term antitumor immune evaluation. A) Schematic of in vivo PDT treatment of bilateral 4T1 tumor‐bearing mice. B) Primary tumor and C) distant tumor growth curves of mice from different groups. Weights of D) primary tumors and E) distant tumor from different groups. F) Survival rates of mice from different groups. G) Flow cytometry analysis of mature DCs (CD80^+^ CD86^+^, gated on CD11c^+^) in lymph nodes. H) Flow cytometry analysis of CD44^+^CD62L^−^ cells in the tumor tissues from different groups. I) Percentage of mDCs, J) percentage of T_EM_ cells, K) percentage of MDSCs cells in spleen and (L) IFN‐γ levels in the primary tumor tissues from different groups. Data presented as mean ± SD, n = 4. p* < 0.05, p** < 0.01, p*** < 0.001, p**** < 0.0001.

Tumor recurrence and metastasis are key contributors to patient mortality post‐treatment. These events can significantly impact the effectiveness of treatments and overall patient prognosis.^[^ ^69^ ^]^ Sustaining a long‐lasting adaptive antitumor immune response is crucial for preventing these outcomes and improving survival rates. Encouraged by the antitumor effect and immune activation effects in 4T1 tumor‐bearing mouse model, we extended our investigation to access the immune efficacy and underlying mechanism in a bilateral 4T1 tumor‐bearing mice model.^[^ ^70^ ^]^ In detail, a primary tumor was inoculated on day ‐7, followed by inoculation of a distant tumor on day ‐3. The mice were randomly divided into six groups once the primary tumor reached ≈80 mm^3^, according to the group categorization (**Figure** **6**A). Treatment was administrated as described in previous section, and only the primary tumors of mice in G4 and G6 groups received light irradiation (0.2 W cm^−3^, 8 min). Similar tumor growth profiles to those in previous section were found for the primary tumors. Encouragingly, these distant tumors without light treatment were also effectively inhibited in G6 group (Figure 6B,C), while the tumor growth in G4 group was only moderately inhibited, due to limited ROS production and insufficient T cell activation. In addition, primary tumor and distant tumor were weighted and as expected, primary tumor weight and distant tumor weight decreased sharply in G6 group (Figure 6D,E), these results corroborated with the tumor growth profiles. Considering that the distant tumors did not receive light irradiation, and excellent inhibition of distance tumors in G6 group clearly indicate the activation of adaptive antitumor immunity. Meanwhile, the survival rate of mice was also largely elongated in G6 group (Figure 6F), where all mice in G1, G2, G3, and G5 groups died within 32 days, mice in G4 group dies within 45 days, whereas all mice survived over 40 days and 3 out of 5 mice still live at day 60. This remarkable difference in tumor inhibition and survival rate confirmed the powerful long‐lasting antitumor effect delivered by BSA@TPE‐BT‐SCP NPs with irradiation.

It has been reported that in antitumor immune responses, mDCs in the draining lymph nodes play a crucial bridging role. The mDCs in tumor‐draining lymph nodes was then accessed. Notably, the population of CD80^+^CD86^+^ mature DCs was significantly increased in BSA@TPE‐BT‐SCP NPs with irradiation group (G6), which was 1.75‐fold higher than Saline control group, and was also slightly higher than G4 group (Figure 6G,I). Effector memory T (T_EM_) cells, when stimulated by the same tumor antigens, can produce antitumor cytokines that directly kill tumor cells.^[^ ^71^ ^]^ The levels of T_EM_ cells in spleen were further evaluated. BSA@TPE‐BT‐SCP NPs + L group (G6) exhibited the highest T_EM_ cell level of 29.87%, which was 1.32‐fold and 1.45‐fold higher than PEG@TPE‐BT‐SCP NPs + Light group (G4) and Saline group (G1) (Figure 6H,J). Intriguingly, we found that the percentage of MDSCs in spleen was decreased from 47% in Saline group to 25% in G4 group, while the MDSCs levels in G6 group (21.5%) were only ≈0.45‐fold of Saline group (Figure 6K; Figure S29, Supporting Information). MDSCs were immature myeloid cells with immunosuppressive effects, which not only promote the tumor invasion and immune escape, but also impeded the antitumor efficacies of PDT and chemotherapy.^[^ ^72^ ^]^ In this regard, BSA@TPE‐BT‐SCP NPs with the capability to downregulate MDSC levels provide additional benefits in remodulating tumor microenvironment for tumor treatment. In addition, the levels of INF‐γ in distant tumor were also significantly increased to nearly 150 pg/mL in G6 group, in sharp contrast to other groups (Figure 6L), further proving the enhanced tumor immunogenicity brought by BSA@TPE‐BT‐SCP NPs.

**Figure 7:**
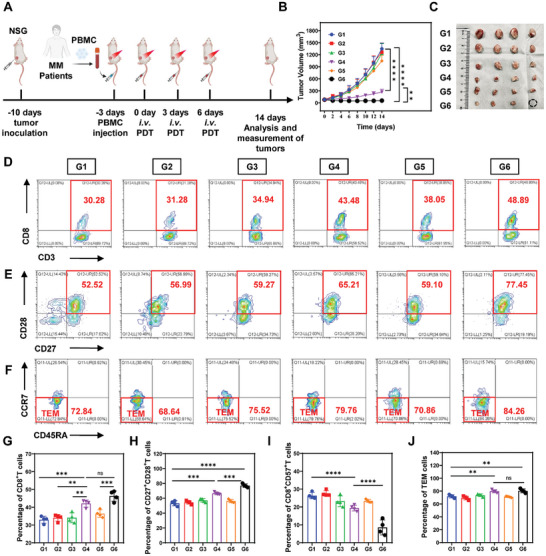
Tumor inhibition in humanized mice model. A) Schematic of in vivo PDT treatment of MM tumor‐bearing NSG mice. B) Comparison of tumor growth over time among different experimental groups. C) Photographs of extracted tumors from different groups. D) Flow cytometry analysis of CD8^+^ CD3^+^ T cells in tumors from different groups. E) Flow cytometry analysis of CD27^+^CD28^+^ T cells in the tumor tissues from different groups. F) Flow cytometry analysis of CD45RA^−^CCR7^−^ cells in the tumor tissues. G) Percentage of CD8^+^ T cells, H) CD27^+^CD28^+^ T cells, I) CD8^+^CD57^+^ T cells, and J) T_EM_ cells in the tumor tissues. Data presented as mean ± SD, n = 4. p* < 0.05, p** < 0.01, p*** < 0.001, p**** < 0.0001.

The excellent tumor inhibition and immune activation properties of BSA@TPE‐BT‐SCP NPs prompted us to evaluate their antitumor effect and clinical immune response in a humanized mouse model.^[^ ^42^ ^]^ To establish a humanized multiple myeloma (MM) tumor model, RPMI‐8266 cells were subcutaneously injected into NSG mice, which lack T cells, B cells, and NK cells. Subsequently, peripheral blood mononuclear cells (PBMCs) from 13 MM patients were intravenously injected into these NSG mice bearing tumors to establish a humanized immune system (see **Figure** **7**A). This study was conducted under the supervision of the Tianjin Medical University General Hospital Ethics Committee. All the patients are duly acknowledged and consented to this study. This model allowed us to simulate the immune responses of MM patients induced by BSA@TPE‐BT‐SCP NPs. When the tumor volumes reached ≈80 mm^3^, the mice were grouped into six categories, according to previous settings, and treated as described previously. As depicted in Figure 7B, tumor volumes in G1, G2, G3, and G5 groups exhibited rapid growth, reaching nearly 1100–1300 mm^3^ at day 14. In contrast, tumor volume in G4 group was moderately inhibited, with a shrunken tumor volume of 270 mm^3^. Intriguingly, BSA@TPE‐BT‐SCP NPs with irradiation (G6) almost completely inhibited tumor growth (Figure 7B,C; Figure S30, Supporting Information). Additionally, body weights of mice in all groups demonstrated the good biocompatibility of these nanoparticles (Figure S31, Supporting Information). To further investigate the regulatory effects of type I ROS on immune activation, the most crucial T cells involved in antitumor immunity was examined (Figure 7D,G). As expected, the G6 group exhibited the highest infiltration of CD8^+^ T cells into the tumor. Due to the immunosuppressive microenvironment within tumors, the cytotoxic effects of T cells on tumors are often inhibited, we thereby conducted an in‐depth investigation of T cell functions in the tumor immune microenvironment. CD8^+^CD27^+^CD28^+^ T cells, indicative of a functional and responsive T cell population capable of effective antitumor responses, can promote a more robust and sustained immune response against tumor cells. As shown in Figure 7E,H, in the G6 group, there was a notably higher percentage of activated CD8^+^CD27^+^CD28^+^ T cells compared to the other groups. On the other hand, the level of CD8^+^CD57^+^ T cells, a senescent subset of T cells that signify a state of T cell immune exhaustion, was significantly reduced in G6 group, suggesting that these activated T cells could function effectively to eliminate cancer cells (Figure 7I; Figure S32, Supporting Information). Furthermore, the percentage of effector memory T cells (TEM, CD3^+^CD8^+^CD45RA^−^CCR7^−^) in the peripheral blood in G6 group was also much higher than those in other groups, proving long‐lasting antitumor immunity (Figure 7J).^[^ ^73^ ^]^ Collectively, BSA@TPE‐BT‐SCP NPs mediated photo‐immunotherapy is capable for provoking a robust and sustained antitumor immune response.

## 3 Conclusion

In this study, we designed a supramolecular PS BSA@TPE‐BT‐SCP NPs, developed from loading AIE PSs into the hydrophobic pocket of BSA protein, to promote type I ROS generation for photo‐immunotherapy. We demonstrated that BSA encapsulation represents a more effective strategy than conventional polymeric matrix encapsulation in promoting type I ROS production, and BSA@TPE‐BT‐SCP NPs exhibited a nearly 52‐fold higher ROS generation capability over PEG@TPE‐BT‐SCP NPs. We revealed that BSA@TPE‐BT‐SCP NPs, through potent type I ROS generation, induced multiple forms of programmed tumor cell deaths, including apoptosis, pyroptosis, and ferroptosis. These multifaceted cell deaths led to the remarkable release of substantial amounts of DAMPs such as HMGB1, HSP70, LDH, and ATP, as well as antitumor cytokines. Both in vitro and in vivo experiments confirmed that BSA@TPE‐BT‐SCP NPs effectively induced DC maturation and T cell activation, increased activated T cell infiltration, and reduced the proportion of senescent T cells, thereby inhibiting both primary and distant tumors. BSA@TPE‐BT‐SCP NPs also exhibited excellent antitumor performance in a humanized mice model, which significantly downgraded the population of senescent T cells among these activated T cells, opening up new possibilities for T cell‐based immunotherapy. These findings not only advance the development of type I PSs but also provide new insights into modulating ROS types to influence tumor cell death and enhance antitumor immunotherapy.

## 4 Experimental Section

### Cells and Animals

4T1 cells were purchased from the ATCC. As for the animal studies, NSG mice were obtained from Shanghai MODEL Organisms for this experiment. Animal experiments were approved by the Animal Ethics Committee of Nankai University and performed by the guidelines SYXK (Jin) 2019‐0003 of Tianjin Experimental Animal Use and Care Committee. Additionally, a study involving 13 newly diagnosed multiple myeloma (MM) patients was conducted at the Hematology Department of Tianjin Medical University General Hospital from June 2022 to February 2023. The study received approval from the Tianjin Medical University General Hospital Ethics Committee (Ethical No. IRB‐ZD‐004(F)‐002‐02). All patients met the diagnostic criteria established by the International Myeloma Working Group (IMWG).

### Statistical Analysis

Statistical analyses included unpaired Student's *t*‐tests for comparing two groups and one‐way ANOVA followed by Tukey's post‐hoc analysis for multiple group comparisons. Graphs were created using GraphPad Prism 8.0.2 software. Results are reported as mean ± standard deviation (SD), with n = 4, unless otherwise specified. Statistical significance was defined as *p* < 0.05 (*p < 0.05, **p < 0.01, ***p < 0.001, ****p < 0.0001).

## Conflict of Interest

The authors declare no conflict of interest.

## Supporting information



Supporting Information

## Data Availability

The data that support the findings of this study are available in the supplementary material of this article.
