# Supplementary material for: An Albumin‐Photosensitizer Supramolecular Assembly with Type I ROS‐Induced Multifaceted Tumor Cell Deaths for Photodynamic Immunotherapy
Source: Adv Sci (Weinh). 2025 Jan 13;12(9):2410405. doi: 10.1002/advs.202410405 (PMC11884554; doi:10.1002/advs.202410405)
Supplement: Supplementary file 1 — Supporting Information [file ADVS-12-2410405-s001.docx]

Supporting Information

An Albumin-Photosensitizer Supramolecular Assembly with Type I ROS-Induced Multifaceted Tumor Cell Deaths for Photodynamic Immunotherapy

*Jingtian Zhang,^#^ Di Jiao,^#^ Xinwen Qi, Yufan Zhang, Xiaoang Liu, Tengwu Pan, Heqi Gao, Zhaoyun Liu,^*^ Dan Ding,^*^ Guangxue Feng^*^*

**This PDF file includes:**

Figs. S1 to S32

**Materials and Methods**

**Materials**

All chemicals and reagents were commercially sourced. DMEM medium, FBS, penicillin-streptomycin, primary antibodies, and fluorescent secondary antibodies for confocal imaging were obtained from Gibco, Abcam, and Invitrogen, respectively. Antibodies were obtained from Abcam or CST. TEMP and DMPO were obtain from DOJINDO (Japan). ELISA kit purchased from Solarbio. LDH release assay kit and ATP assay kit were purchased from Beyondtime.

**Computational method**

The Gaussian 09 program (Revision D01) was employed for density functional theory (DFT) calculations. Optimization of ground state (S_0_) geometries was conducted using the B3LYP/6-31G (d) method.

**Synthesis of compound 1**

In a reaction vessel, 4,4'-(2-(4-bromophenyl)-2-phenylethene-1,1-diyl)bis(methoxybenzene) (471 mg, 1 mmol), bis(pinacolato)diboron (303 mg, 1.2 mmol), and potassium acetate (294 mg, 3 mmol) were dissolved in 5 mL of acetic acid. The mixture was heated at 110 °C under a nitrogen atmosphere for 8 hours and then subjected to vacuum. After completion, the crude product was obtained by vacuum drying, followed by purification using silica gel column chromatography, resulting in a white solid (478 mg, 92.3% yield).^1^H NMR (400 MHz, Chloroform-d) δ 7.56 – 7.51 (m, 2H), 7.09 – 7.07 (m, 2H), 7.03 (s, 1H), 7.02 – 6.97 (m, 3H), 6.94 (d, J = 1.5 Hz, 2H), 6.92 (d, J = 1.5 Hz, 2H), 6.62 (dd, J = 8.8, 1.4 Hz, 4H), 3.74 (d, J = 2.6 Hz, 6H), 1.32 (s, 12H).

**Synthesis of compound 2**

Compound 1 (478 mg, 0.92 mmol), 7-bromobenzo[c] [1,2,5] thiadiazole-4-carbaldehyde (266 mg, 1.1 mmol), and PdPPh_3_ (11 mg, 0.009 mmol) were dissolved in 8 mL of THF, and then added 2 mM K_2_CO_3_ of aqueous solution (6 mL). The reaction was heated to 75 °C under N_2_ atmosphere for 8 h. After completion of the reaction, the solution was diluted with EA and extracted three times with H_2_O. The organic phase was collected and the crude product was obtained by drying under vacuum. A yellow solid (459 mg, 89 % yield) was obtained by silica gel column chromatography, dried under vacuum.^1^H NMR (400 MHz, Chloroform-*d*) δ 10.76 (s, 1H), 8.27 (d, *J* = 7.4 Hz, 1H), 7.86 (d, *J* = 7.4 Hz, 1H), 7.83 – 7.79 (m, 2H), 7.23 – 7.20 (m, 2H), 7.15 – 7.09 (m, 5H), 7.04 – 7.01 (m, 2H), 6.96 (d, *J* = 8.7 Hz, 2H), 6.66 (dd, *J* = 11.0, 8.7 Hz, 5H), 3.74 (d, *J* = 2.8 Hz, 6H).

**Synthesis of compound 3**

2-(4-bromophenyl) acetonitrile (388 mg, 2 mmol), 4-(4,4,5,5-tetramethyl-1,3,2-dioxaborolan-2-yl) pyridine (410 mg, 2 mmol), PdPPh_3_ (23 mg, 0.02 mmol) were dissolved in 5 mL of THF. Then, 4 mL of 2 mM K_2_CO_3_ aqueous solution was added. the reaction was heated to 75 °C under N_2_ atmosphere for 8 h. When the reaction process was finished, the reaction was diluted by adding EA and extracted three times with H2O. The organic phase was collected and dried under vacuum to obtain the crude product. Further purification by silica gel column gave white solid compound 3(349 mg, 90% yield). ^1^H NMR (400 MHz, Chloroform-*d*) δ 8.73 – 8.65 (m, 2H), 7.69 – 7.64 (m, 2H), 7.53 – 7.45 (m, 4H), 3.83 (s, 2H).

**Synthesis of TPE-BT-SCP**

Compound 2 (459 mg, 0.83 mmol), compound 3 (193 mg, 0.99 mmol), and EtONa (56 mg, 0.83 mmol) were dissolved in 5 mL of ethanol. The reaction mixture was heated to 80 °C under a nitrogen atmosphere. After 6 hours, the solvent was removed under vacuum, yielding an orange solid crude product. This solid was dissolved in 5 mL of acetonitrile (CAN), and iodomethane (589 mg, 4.15 mmol) was added. The reaction continued at 80 °C under nitrogen for another 6 hours. After completion, purification by silica gel column chromatography afforded a red solid. This red solid was dissolved in 10 mL of acetone, followed by addition of 10 mL of saturated aqueous KPF6 solution. The mixture was stirred at room temperature for 2 hours. After extraction with dichloromethane three times, the red solid TPE-BT-SCP was obtained by vacuum drying (632 mg, 86% yield). HRMS (C_49_H_37_N_4_O_2_S): m/z [M]^-^calcd. 745.2632, found 745.2625. ^1^H NMR (400 MHz, DMSO-d6) δ 9.07 (d, J = 6.4 Hz, 2H), 8.73 (d, J = 4.0 Hz, 1H), 8.58 (dd, J = 7.3, 3.5 Hz, 3H), 8.28 (d, J = 8.1 Hz, 2H), 8.08 (d, J = 7.8 Hz, 3H), 7.93 (d, J = 8.0 Hz, 2H), 7.16 (dq, J = 15.5, 7.5 Hz, 5H), 7.00 (dd, J = 21.1, 7.8 Hz, 4H), 6.89 (d, J = 8.2 Hz, 2H), 6.73 (dd, J = 21.8, 8.3 Hz, 4H), 4.35 (s, 3H), 3.69 (s, 6H). ^13^C NMR (101 MHz, DMSO-d6) δ 158.42, 158.29, 154.57, 153.38, 152.74, 146.23, 145.29, 144.20, 141.12, 138.59, 138.38, 137.10, 136.04, 135.24, 134.83, 134.21, 132.62, 132.55, 131.62, 131.35, 129.53, 129.22, 129.08, 128.48, 128.13, 127.54, 126.90, 125.54, 124.61, 117.76, 113.88, 113.66, 112.05, 55.40, 47.61.

**Synthesis of BSA@TPE-BT-SCP NPs**

100 mg of BSA powder was added into 50 mL ddH_2_O (50 - 55 ^o^C) to be dissolved completely. Then, 100 μL of dithiothreitol (DTT) solution was added. Subsequently, BSA@TPE-BT-SCP NPs were prepared by adding TPE-BT-SCP (2 mg/mL in 1 mL THF) dropwise into the BSA solution. After a 20-minute reaction, the mixture was cooled to 20 ^o^C using ice-water to obtain the BSA@TPE-BT-SCP NPs.

**Synthesis of PEG@TPE-BT-SCP NPs**

1 mL of THF was utilized to dissolve DEPE-PEG_2000_ (4.0 mg) and TPE-BT-SCP (1.0 mg). The THF solution was then added dropwise into 9.0 mL of deionized water under ultrasound sonication, followed by an additional 3 minutes of sonication. THF was subsequently removed using an air pump for 4 hours in a fume hood. The resulting suspension of PEG@TPE-BT-SCP NPs was dialyzed against deionized water overnight at room temperature using a dialysis membrane with a molecular cutoff of 12 kDa to eliminate any residual THF and excess DSPE-PEG_2000_.

**Cells and animals**

4T1 cells was purchased from the ATCC. As for the animal studies, NSG mice was obtained from Shanghai MODEL Organisms for this experiment. Animal experiments were approved by the Animal Ethics Committee of Nankai University and performed by the guidelines SYXK (Jin) 2019-0003 of Tianjin Experimental Animal Use and Care Committee. Additionally, a study involving 13 newly diagnosed multiple myeloma (MM) patients was conducted at the Hematology Department of Tianjin Medical University General Hospital from June 2022 to February 2023. The study received approval from the Tianjin Medical University General Hospital Ethics Committee (Ethical No. IRB-ZD-004(F)-002-02). All patients met the diagnostic criteria established by the International Myeloma Working Group (IMWG).

**ROS detection assays**

Total ROS were detected using DCFH. To prepare the activated DCFH solution, DCFH-DA powder (500 μg) was dissolved by anhydrous ethanol (1mL) and then added to 4 mL of NaOH solution. After 15 minutes, PBS was added to stop the activation. The activated DCFH solution was mixed with NPs, final TPE-BT-SCP concentration was 5 μM and a final DCFH concentration was 50 μM. Fluorescence emission at 520 nm was measured using a fluorescence spectrophotometer after exposure to white light for designated periods.

Singlet oxygen was detected using the ABDA indicator. 50 mM ABDA was prepared in DMSO and diluted 1:1000 into a 5 μM NPs solution. The mixture was irradiated with light for designated periods, and detected ABDA absorbance at 400 nm.

Hydroxyl radicals were detected with HPF. A mixture of HPF (10 μM) and NPs (5 μM) in 3 mL was exposed to light for designated periods, and HPF fluorescence emission at 520 nm was measured using a fluorescence spectrophotometer.

Superoxide anion generation was detected using DHR123. A mixture of DHR123 (10 μM) and NPs (5 μM) in 3 mL was exposed to light for designated periods, and fluorescence emission at 515 nm was measured using a fluorescence spectrophotometer.

**Detection of O_2_^•−^, •OH and ^1^O_2_ in EPR**

TEMP and DMPO were employed as spin-trapping agents for ^1^O_2_, O_2_^•−^ and •OH, respectively. In the experiment, the solution was mixed with NPs (10 μM based on TPE-BT-SCP). The sample was illuminated with white light for 30 seconds, and the EPR spectra were promptly recorded within 10 minutes.

**Cell viability study**

To evaluate dark toxicity, 5 × 10³ 4T1 cells were incubated with NPs solutions for 24 hours in 96 well plates. Following incubation, washed with PBS, and their viability was tested by the MTT assay. Optical density readings were obtained using a microplate reader to determine cell viability.

To assess phototoxicity, 5 × 10³ 4T1 cells were incubated with NPs solutions for 24 hours in 96 well plates. Subsequently, they were exposed to white light (0.2 W/cm²) for 3 minutes. After light exposure, cells were cultured for another 16 hours, and their viability was tested by the MTT assay. Optical density readings were obtained using a microplate reader to determine cell viability.

**Western blot assay**

Cells from each treatment group were washed with PBS. Following centrifugation (12000 g, 30 minutes), the supernatant was collected and mixed with loading buffer. The protein mixture was then boiled to denature the proteins. The denatured proteins were subsequently electrophoresed and transferred onto membranes. The membranes were first incubated overnight at 4 °C with primary antibodies, followed by a subsequent 1-hour incubation at room temperature with secondary antibodies. Chemiluminescence imaging was used for visualization. The antibodies used included anti-HMGB1 (Abcam, ab79823), anti-HSP70 (Abcam, ab181606), anti-GAPDH (Abcam, ab9485), anti-GPX4 (Abcam, ab125066), anti-GSDMD (Abcam, ab219800), and anti-caspase-1 (E2Z1C, CST 24232).

**Casepase-3 detection and LPO detection assay**

To investigate the mechanism of cell death induced by NPs and RSL3, 4T1 cells were cultured in confocal dishes. Cells were treated with NPs (10 μM TPE-BT-SCP) for 8 hours, followed by exposure to white light (0.2 W/cm^2^, 3 min). After 24 hours, cells were fixed in 4% paraformaldehyde for 20 minutes, and rinsed with PBST. Subsequently, cells were blocked with 3% BSA solution, stained with either caspase-3 antibody (diluted 1:800 in PBST with 1% BSA) or BODIPY581/591-C11 for 1 hour, and visualized using an LSM800 confocal microscope.

**Ecto-CRT staining assay**

4T1 cells were cultured in confocal dishes and allowed to adhere. After 8 hours of incubation with NPs, cells were washed thrice with PBS and exposed to white light (0.2 W/cm2, 3 minutes). Following a 12-hour incubation period, cells were fixed in 4% paraformaldehyde in pre-cooled PBS on ice for 20 minutes. Subsequently, cells were incubated overnight with anti-calreticulin antibody, followed by a 2-hour incubation with a secondary antibody at room temperature. Finally, cells were stained with DAPI and observed using an LSM800 confocal microscope.

***In vivo* 4T1 tumor inhibition study**

Upon subcutaneous injection near the mammary fat pad of mice, 1 × 10^6^ 4T1 cells were administered. The mice were then randomly allocated into six groups once the tumor volume reached 80 mm^3^ : Saline (G1); Saline + Light (G2); PEG@TPE-BT-SCP NPs (G3); PEG@TPE-BT-SCP NPs + Light (G4); BSA@TPE-BT-SCP NPs (G5); BSA@TPE-BT-SCP NPs + Light (G6). The mice in G3, G4, G5, G6 groups received NPs intravenously on days 0, 3 and 6, mice from groups G4 and G6 underwent white light exposure (0.2 W/cm², 8 min) 8 hours post-injection. After 18 days, the mice were euthanized, and the tumor tissues were harvested, weighed, and measured. Each tissue was divided into three parts: one for flow cytometry to analyze immune markers, another for detecting cytokines in the tumor lysate supernatant, and the last for immunofluorescence staining.

**MM tumor inhibition in humanized mice model *in vivo***

The study began by inoculating RPMI8266 cells (2 × 10^7^ cells in 50 μL Matrigel) subcutaneously into mice. After 7 days, the mice were randomly assigned into six groups, similar to the grouping method used for the 4T1 subcutaneous tumor model. PBMCs from MM patients (2 × 10^7^ cells) were then injected into the mice. Treatments were administered on days 0, 3, and 6, followed by photodynamic therapy. On the eighth day, the mice were euthanized, and their tumors were collected, weighed, and measured. Each tumor was sectioned into three parts: one for flow cytometry to analyze immune markers, another for detecting cytokines in the tumor lysate supernatant, and the final part for frozen sections and immunofluorescence staining.

**Immune responses analysis**

At designated time points following treatment, tumors, spleens, and lymph nodes were harvested and processed into single-cell suspensions. Tumors were digested with a collagenase IV and DNase I mixture at 37 °C for 1 hour, then filtered through a 70 μm cell strainer. The resulting single-cell suspensions were stained with flow cytometry antibodies and analyzed using flow cytometry.

In addition to flow cytometry, tumor tissues were subjected to immunofluorescence staining. The tissues were fixed in 4% paraformaldehyde for 48 hours and then dehydrated sequentially through 20%, 30%, and 40% sucrose solutions, with each concentration step lasting 24 hours. The dehydrated tissues were sectioned into 7 μm thick slices using a cryostat. These sections were subsequently stained with specific antibodies and examined using a confocal microscope.

The concentrations of TNF-α and IFN-γ in the tumor tissue supernatant were quantified using ELISA kits according to the manufacturer's instructions.

**Statistical analysis**

Statistical analyses included unpaired Student’s t-tests for comparing two groups and one-way ANOVA followed by Tukey’s post-hoc analysis for multiple group comparisons. Graphs were created using GraphPad Prism 8.0.2 software. Results are reported as mean ± standard deviation (SD), with n = 4, unless otherwise specified. Statistical significance was defined as *p* < 0.05 (**p* < 0.05, ***p* < 0.01, ****p* < 0.001, *****p* < 0.0001).

**Supplementary Figures**

**Figure S1.** Synthetic route of TPE-BT-SCP.


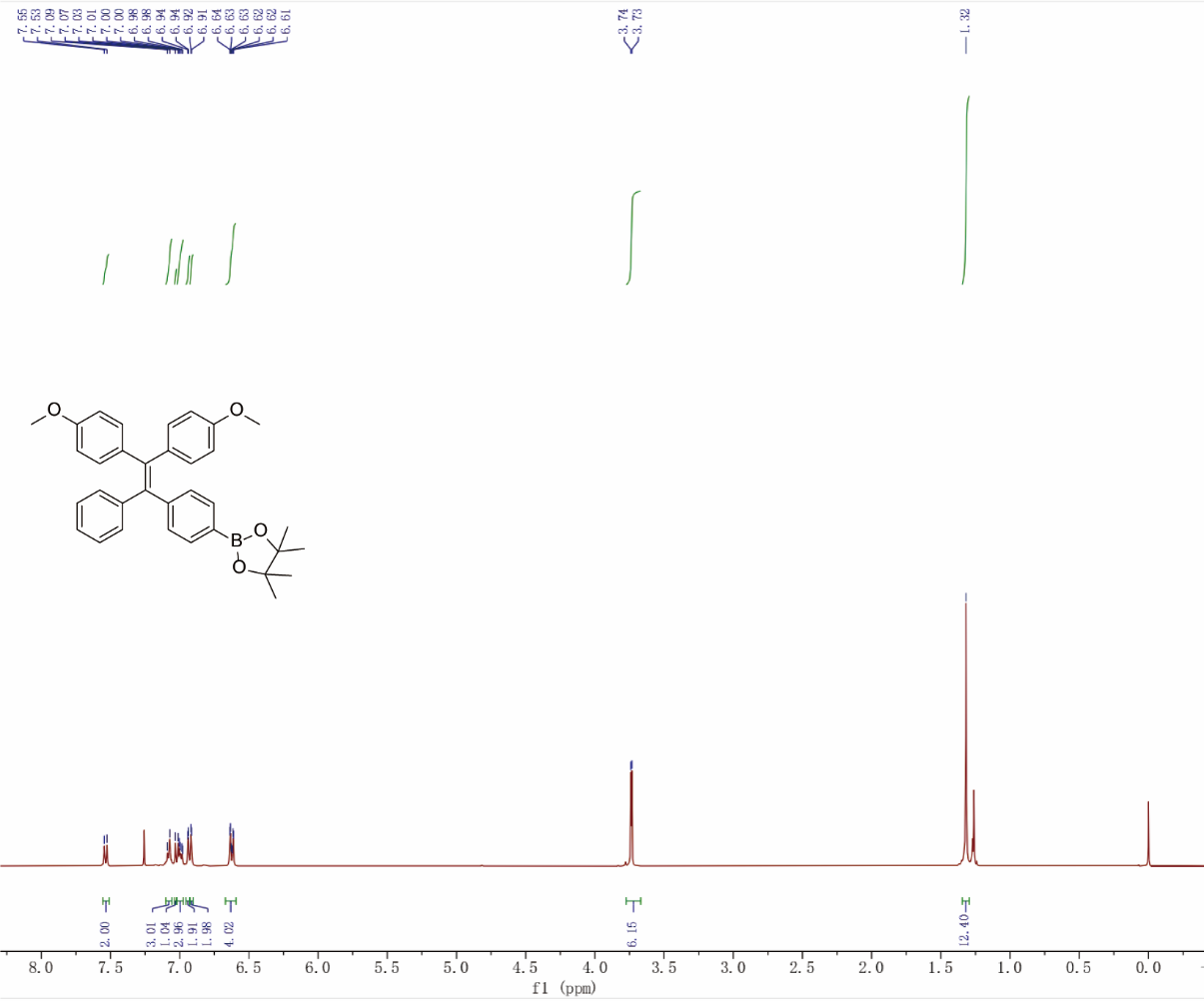


**Figure S2.** ^1^H NMR spectrum of compound 1.


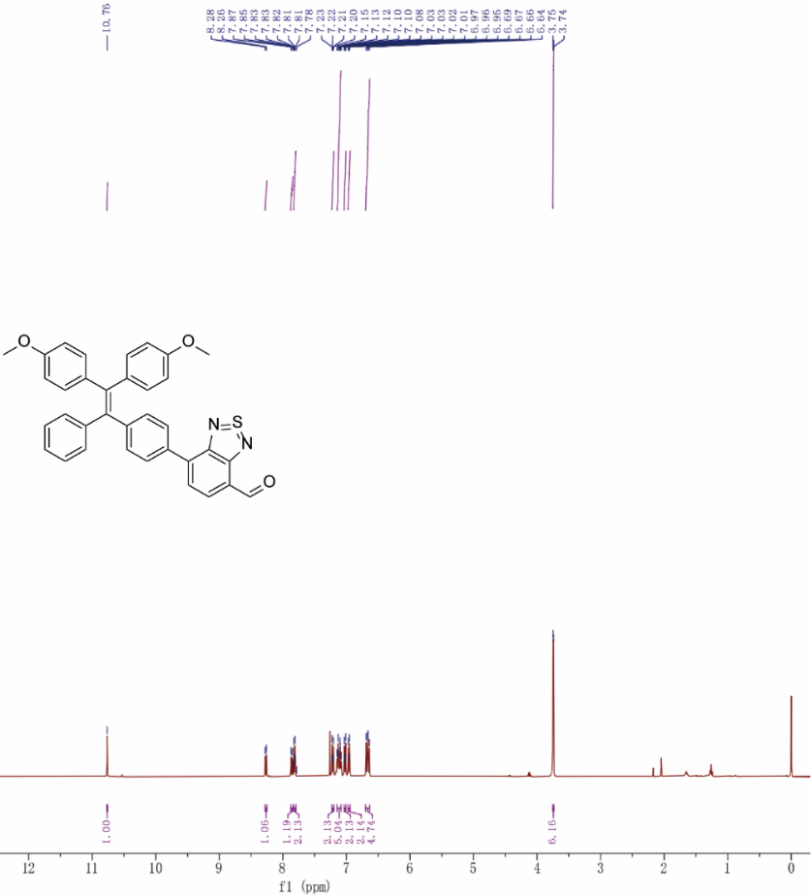


**Figure S3.** ^1^H NMR spectrum of compound 2.


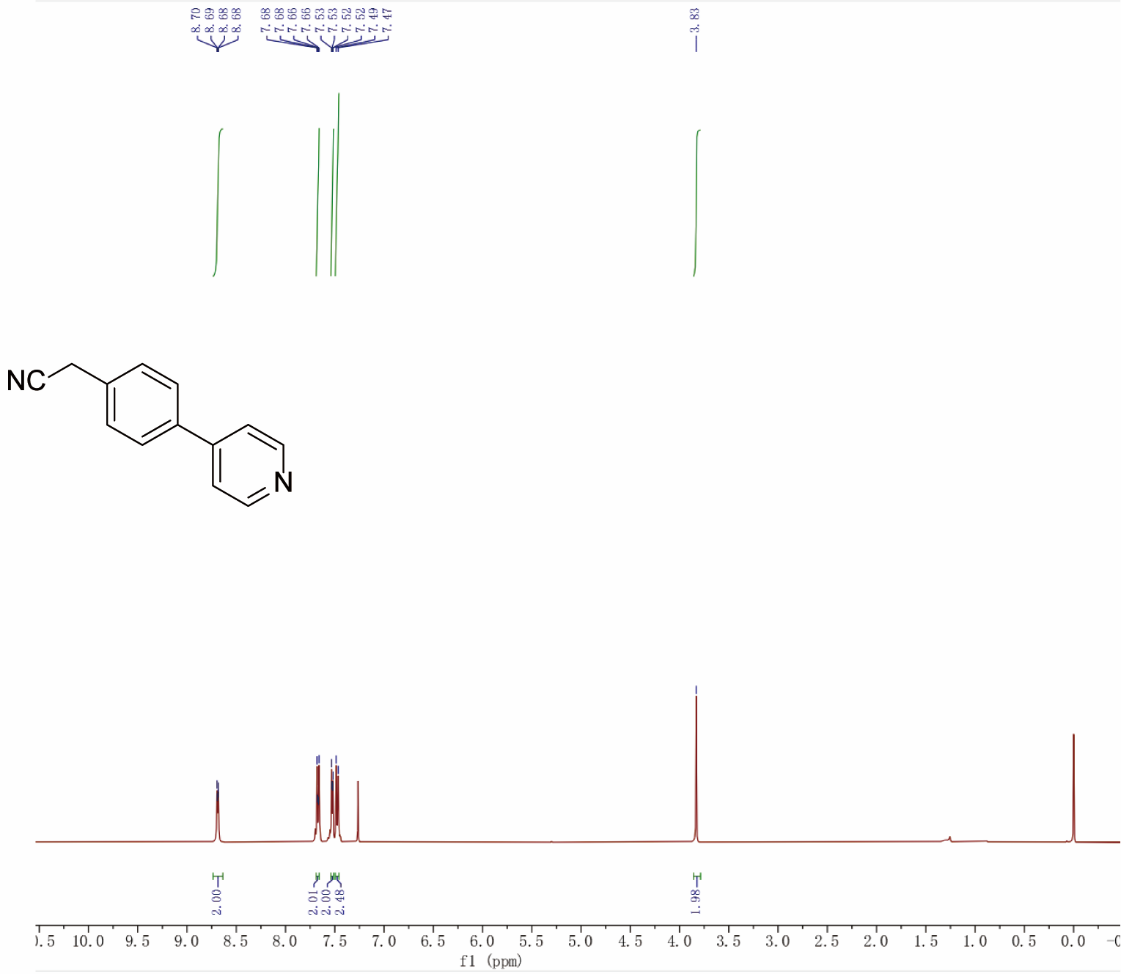


**Figure S4.** ^1^H NMR spectrum of compound 3.


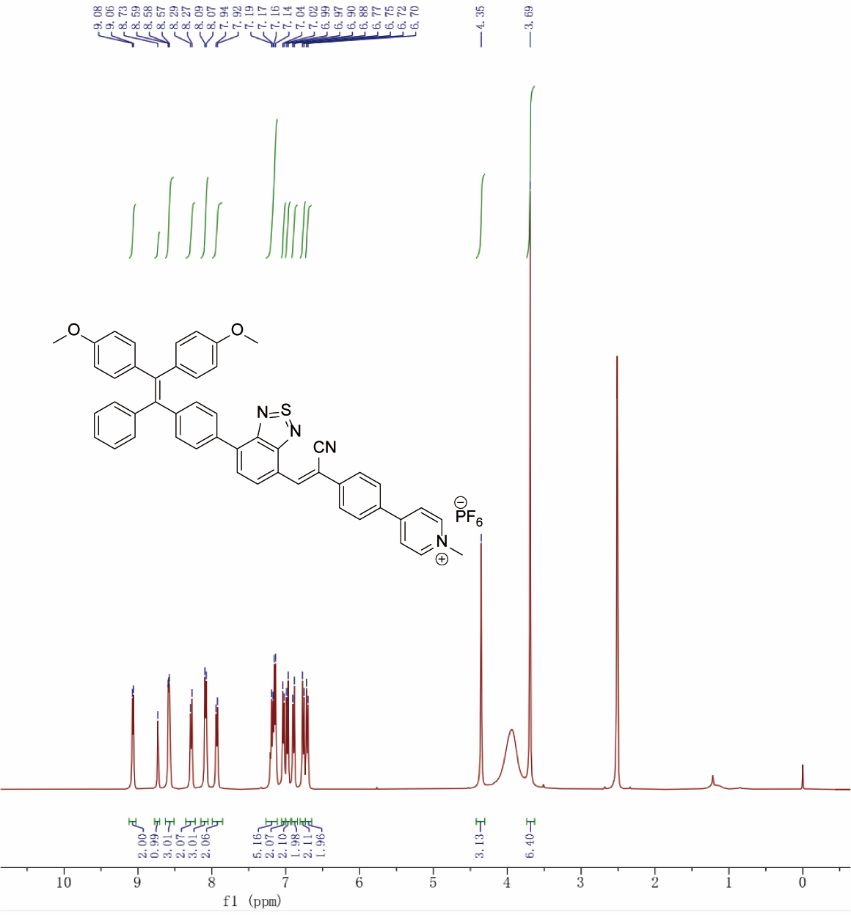


**Figure S5.** ^1^H NMR spectrum of TPE-BT-SCP.


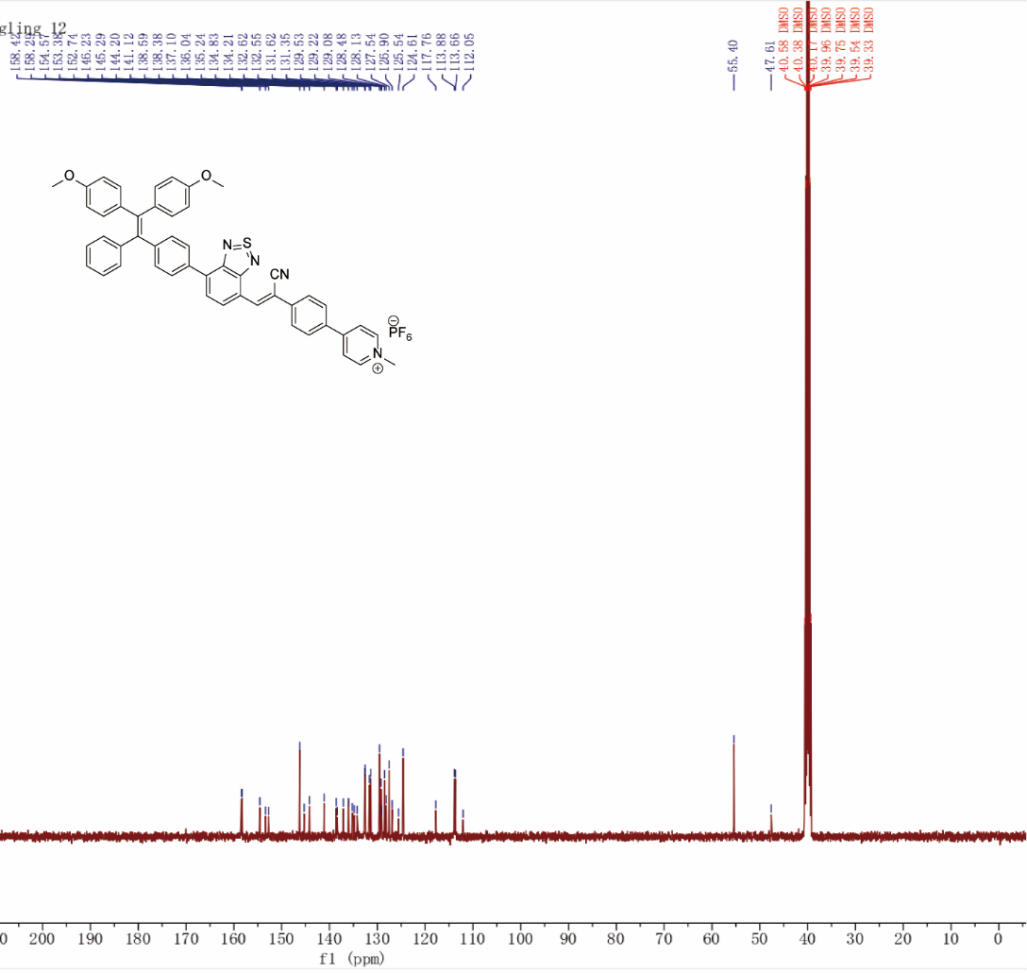


**Figure S6.** ^13^C NMR spectrum of TPE-BT-SCP.


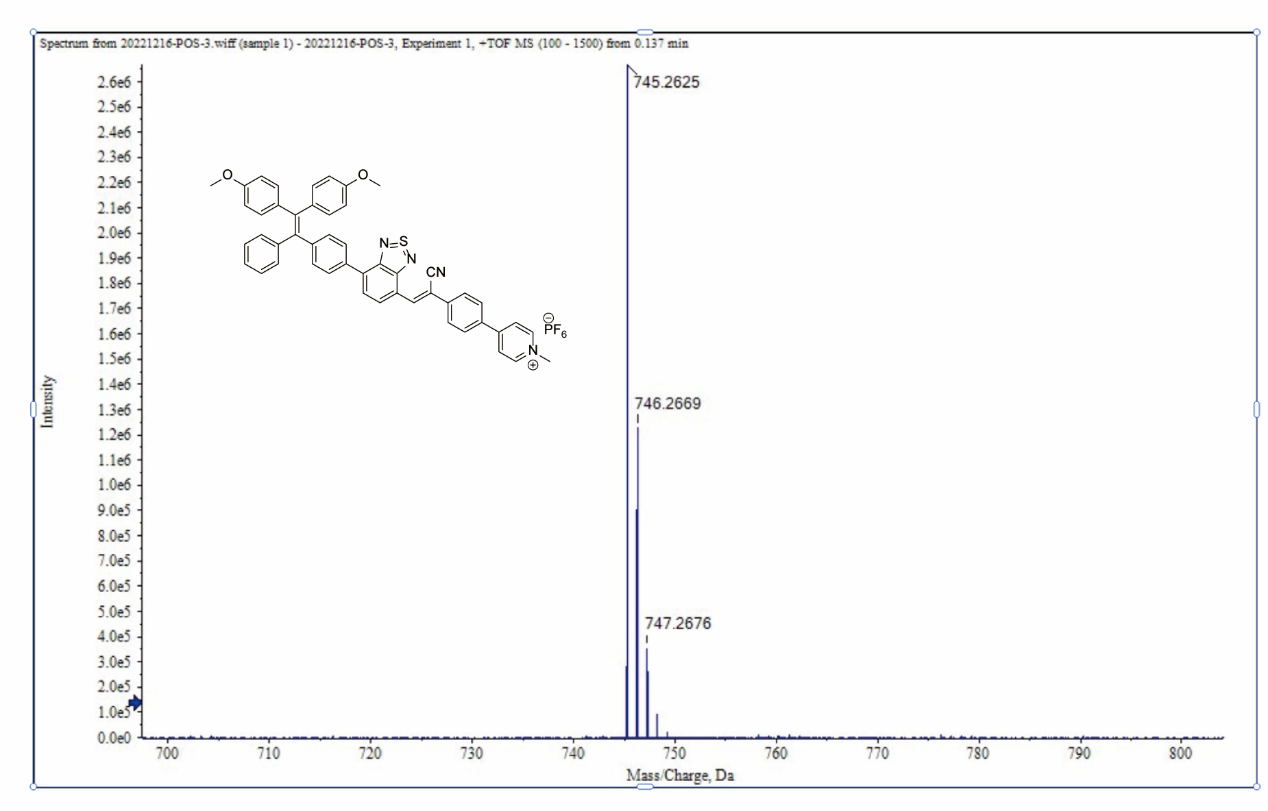


**Figure S7.** HRMS spectrum of TPE-BT-SCP.


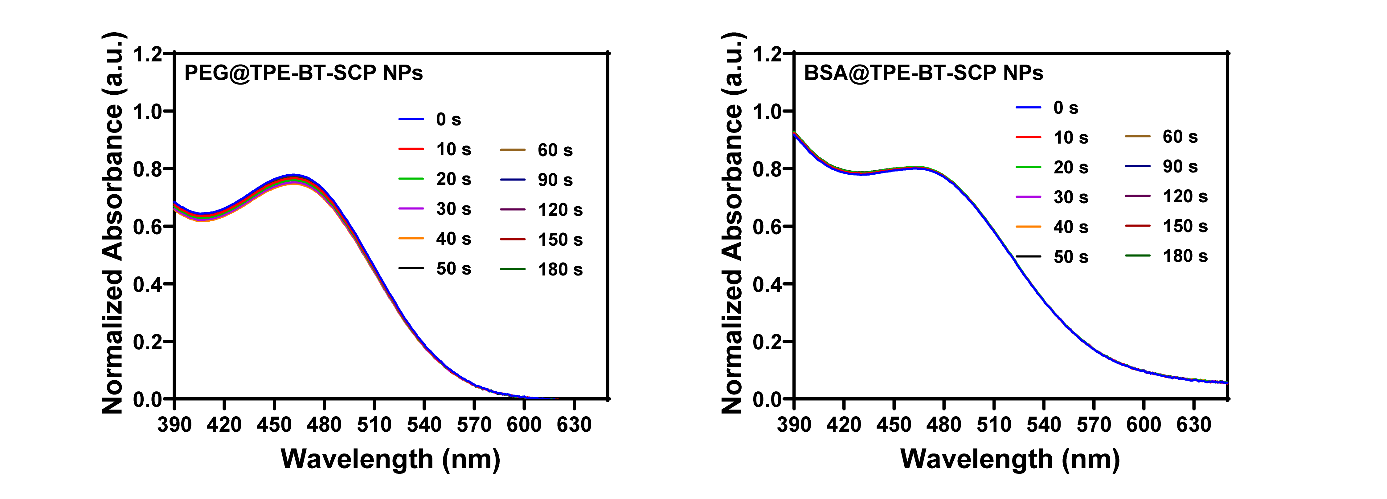


**Figure S8.** The absorbance spectra of PEG@TPE-BT-SCP NPs and BSA@TPE-BT-SCP NPs in PBS solution before and after white light irradiation (0.2 W/cm^2^).


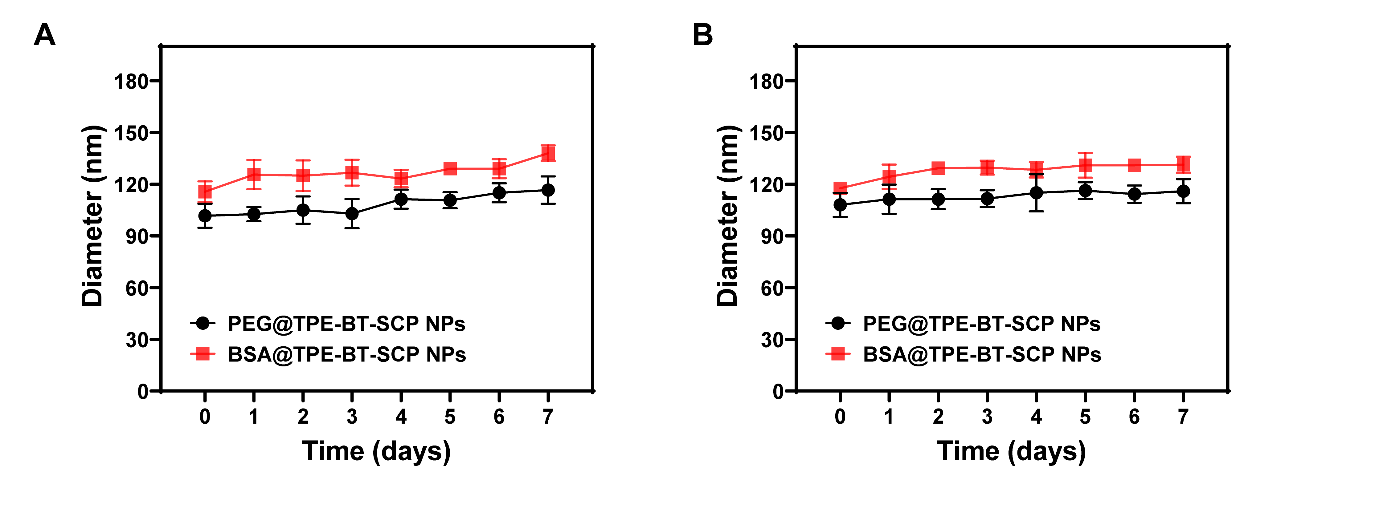


**Figure S9.** The hydrodynamic diameter changes of PEG@TPE-BT-SCP NPs and BSA@TPE-BT-SCP NPs in (A) FBS or (B) in acetate buffer solutions with pH = 5 for 7 days. Data presented as mean ± SD, n = 3.


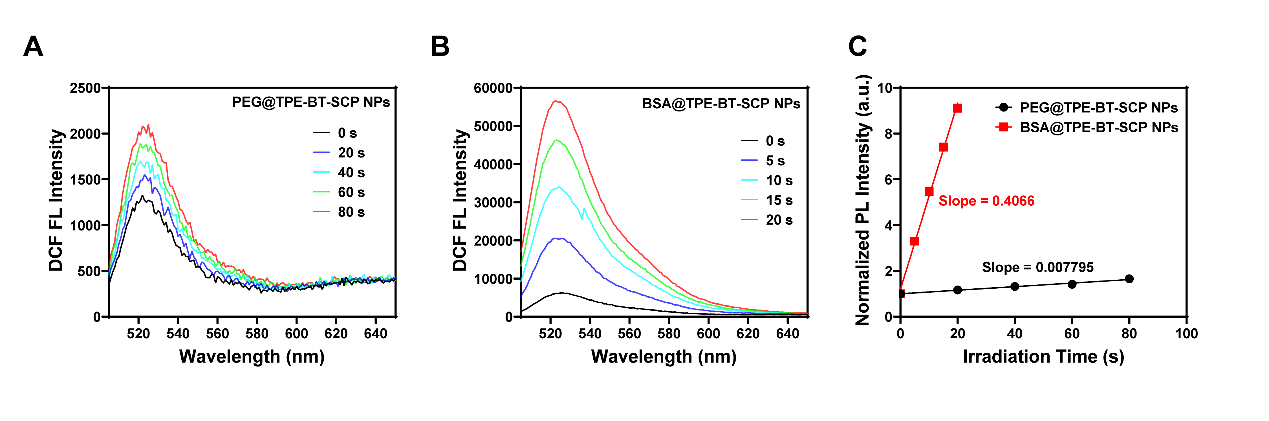


**Figure S10.** PL spectra changes of DCF in the presence of (A) PEG@TPE-BT-SCP NPs and (B) BSA@TPE-BT-SCP NPs, upon white light irradiation (0.2 W/cm^2^, NPs concentration: 5 μM). (C) Slope of PL intensity of DCF changes.


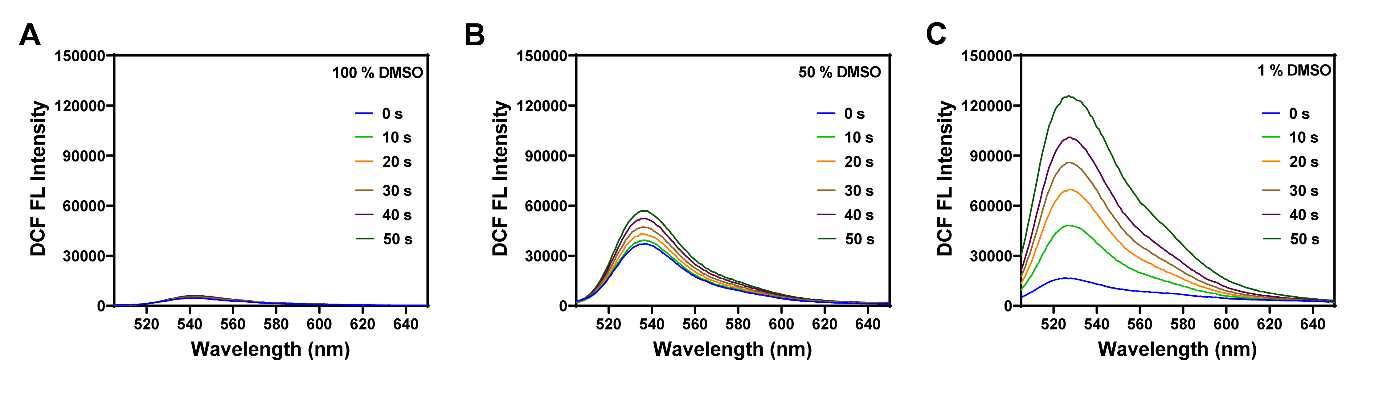


**Figure S11.** PL spectrum changes of DCF in the presence of TPE-BT-SCP (5 μM) in in DMSO/Tol mixture with (A) 100 %, (B) 50 %, and (C) 1 % DMSO fractions, under white light irradiation (0.2 W/cm^2^).


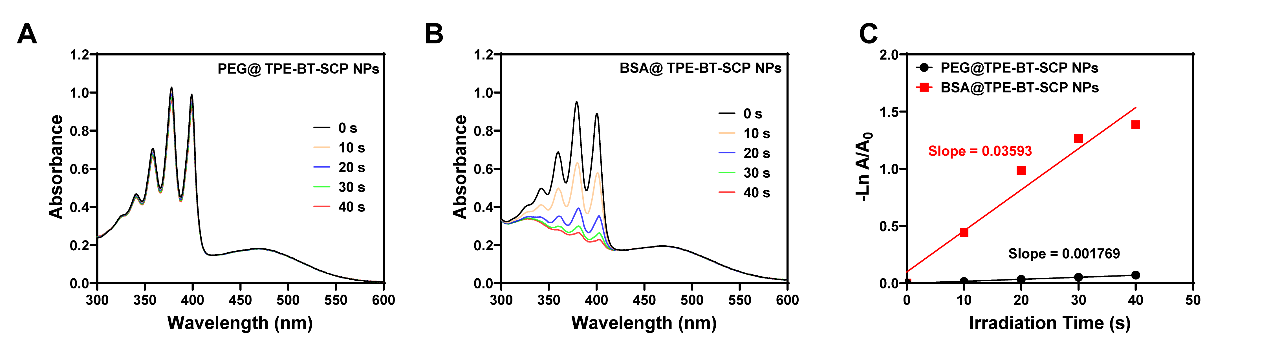


**Figure S12.** Absorption spectra changes of ABDA in the presence of (A) PEG@TPE-BT-SCP NPs and (B) BSA@TPE-BT-SCP NPs, upon white light exposure (0.2 W/cm^2^, NPs concentration: 5 μM). (C) Plot of –ln(A/A_0_) versus light exposure time with different nanoparticles, where A_0_ and A represent the ABDA absorbance before and after irradiation, respectively.


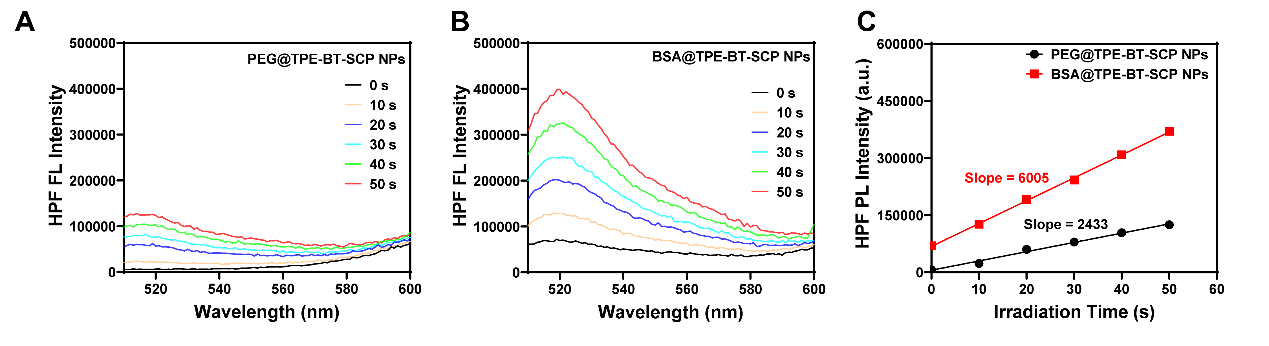


**Figure S13.** PL spectra changes of HPF in the presence of (A) PEG@TPE-BT-SCP NPs and (B) BSA@TPE-BT-SCP NPs, upon white light irradiation (0.2 W/cm^2^, NPs concentration: 5 μM). (C) Slope of PL intensity of HPF for both nanoparticles versus irradiation time.


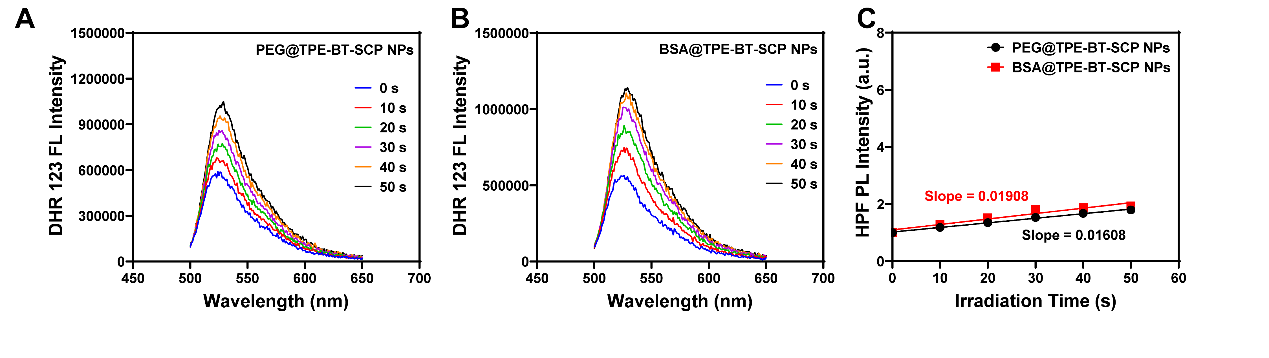


**Figure S14.** PL spectra changes of DHR123 in the presence of (A) PEG@TPE-BT-SCP NPs and (B) BSA@TPE-BT-SCP NPs, upon white light irradiation (0.2 W/cm^2^, NPs concentration: 5 μM). (C) Slope of PL intensity changes of DHR123.


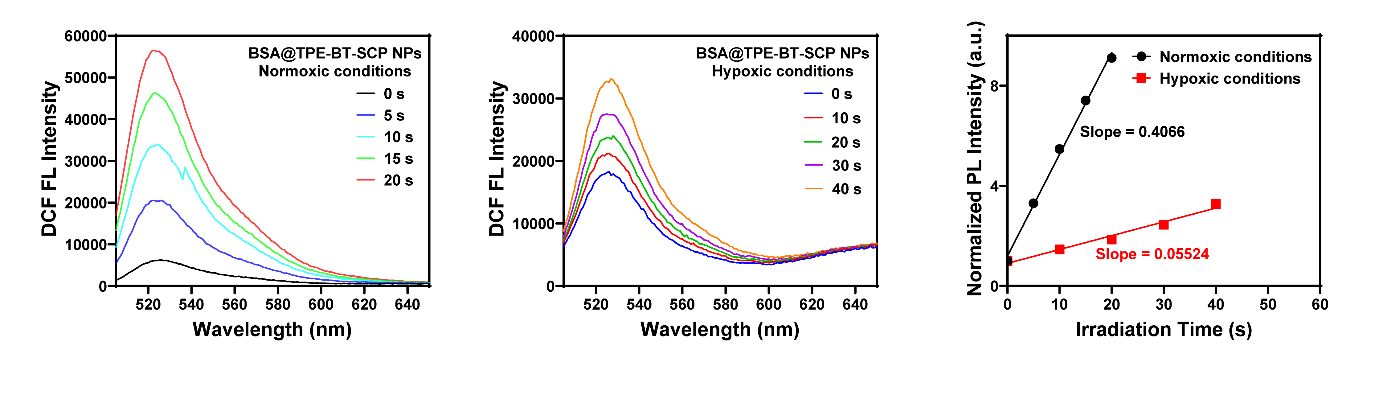


**Figure S15.** PL spectra changes of DCF in the presence of BSA@TPE-BT-SCP NPs upon white light irradiation, under normoxic conditions and hypoxic conditions.


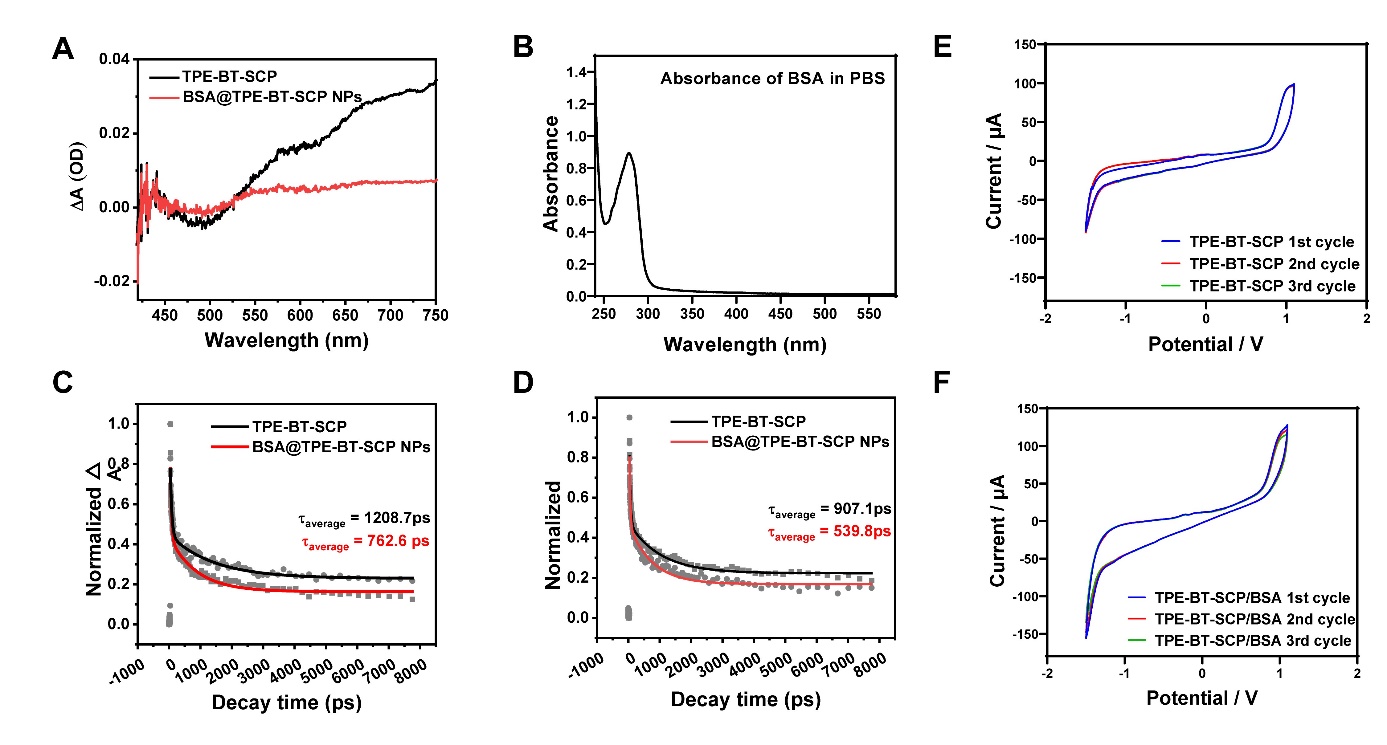


**Figure S16.** (A) Transient absorbance spectra of TPE-BT-SCP and BSA@TPE-BT-SCP NPs in aqueous solution under anaerobic conditions by argon bubbling. (B) Absorbance spectrum of BSA. (C) Kinetic fitting of ESA band (at 725 nm) of TPE-BT-SCP and BSA@TPE-BT-SCP under anaerobic conditions. (D) Kinetic fitting of ESA-normalized TPE-BT-SCP and BSA@TPE-BT-SCP at 725 min under air conditions. (E) Three cycles of cyclic voltammograms of TPE-BT-SCP and (F) BSA@TPE-BT-SCP NPs in nitrogen-saturated PBS containing 20% DMF in volume, which were recorded at 0.05 V/s using a three-electrode system (all potentials were referenced to NHE via an external ferrocene calibration).


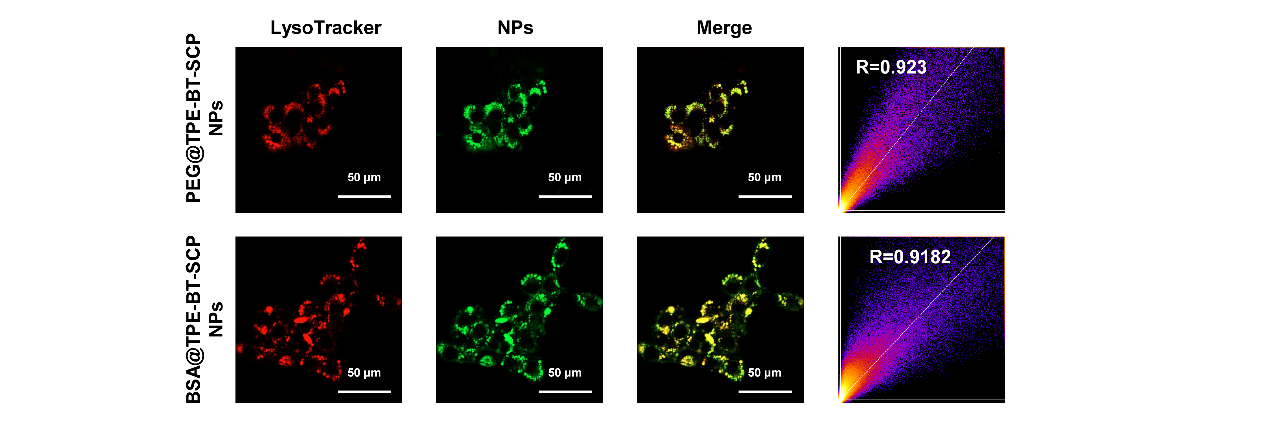


**Figure S17.** Lysosome colocalization of PEG@TPE-BT-SCP NPs or BSA@TPE-BT-SCP NPs in 4T1 cells after incubation at 37 °C for 8 h, respectively. Red: lysosomes; green: TPE-BT-SCP. Scale bars: 50 μm.


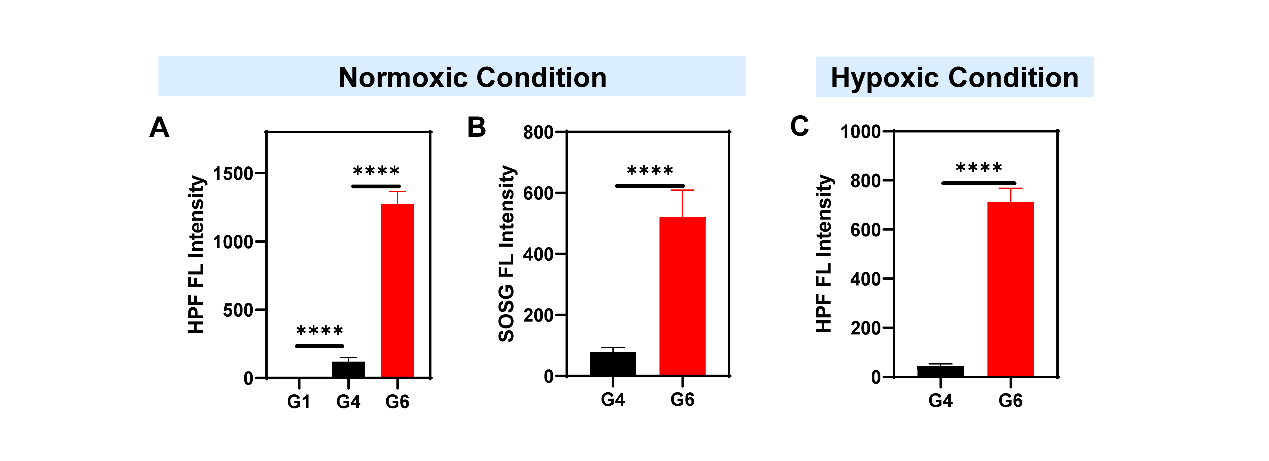


**Figure S18.** Quantitative data showing the mean fluorescence intensity (MFI) of (A) DCF in normoxic condition, (B) SOSG in normoxic condition and (C) HPF in hypoxic condition. According to the representative images in Figure 3A. Error bars: mean ± SD (n = 3). **p* < 0.05, ***p* < 0.01, ****p* < 0.001, *****p* < 0.0001.


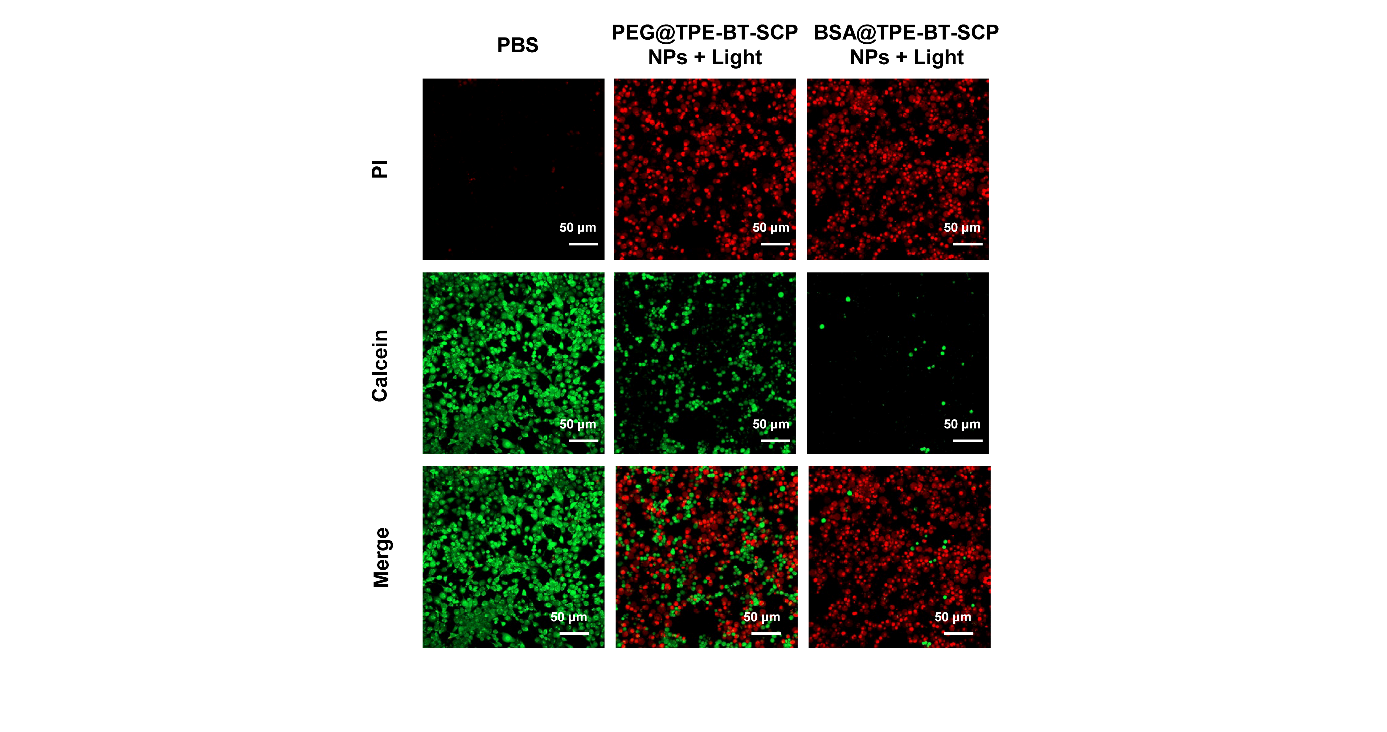


**Figure S19.** CLSM evaluation of tumor killing effect of PEG@TPE-BT-SCP NPs (5 μM) and BSA@TPE-BT-SCP (5μm) in 4T1 cells under white light irradiation (0.2 W/cm^2^) with Calcein AM/PI assay.


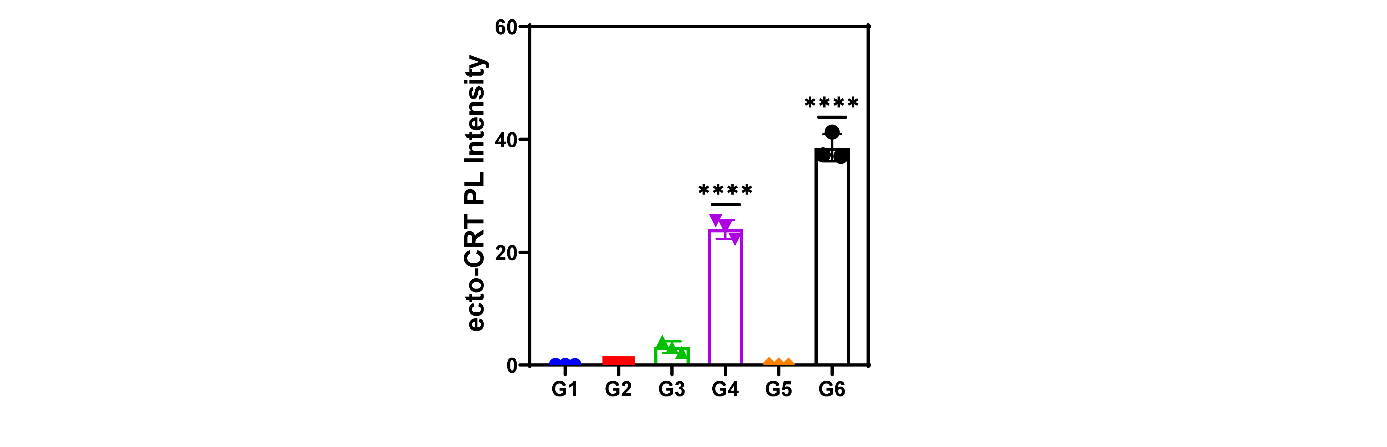


**Figure S20.** Quantitative data showing the mean fluorescence intensity (MFI) of ecto-CRT. According to the representative images in Figure 3E. Error bars: mean ± SD (n = 3). **p* < 0.05, ***p* < 0.01, ****p* < 0.001, *****p* < 0.0001.


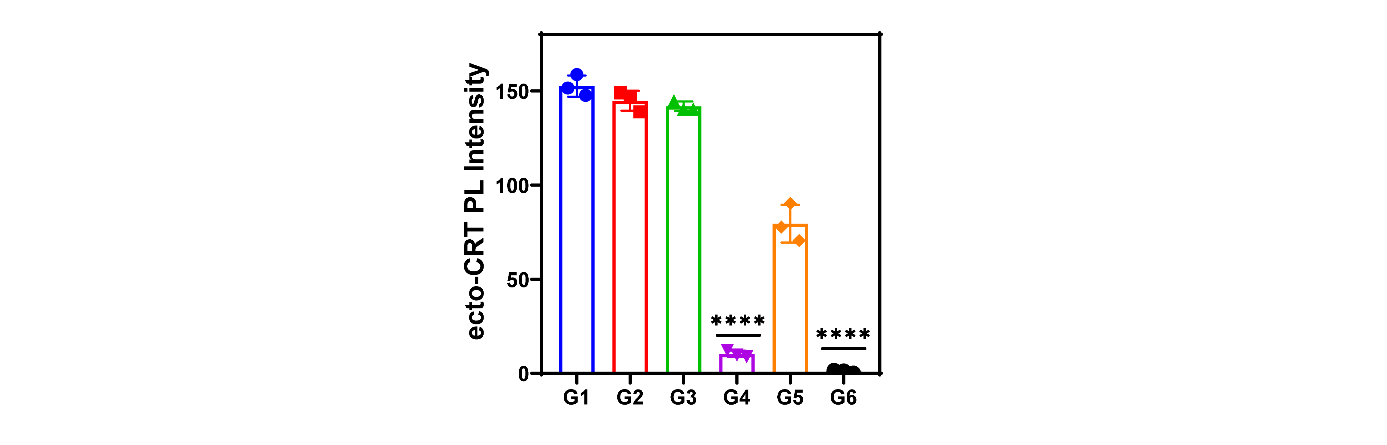


**Figure S21.** Quantitative data showing the mean fluorescence intensity (MFI) of HMGB1. According to the representative images in Figure 3F. Error bars: mean ± SD (n = 3). **p* < 0.05, ***p* < 0.01, ****p* < 0.001, *****p* < 0.0001.


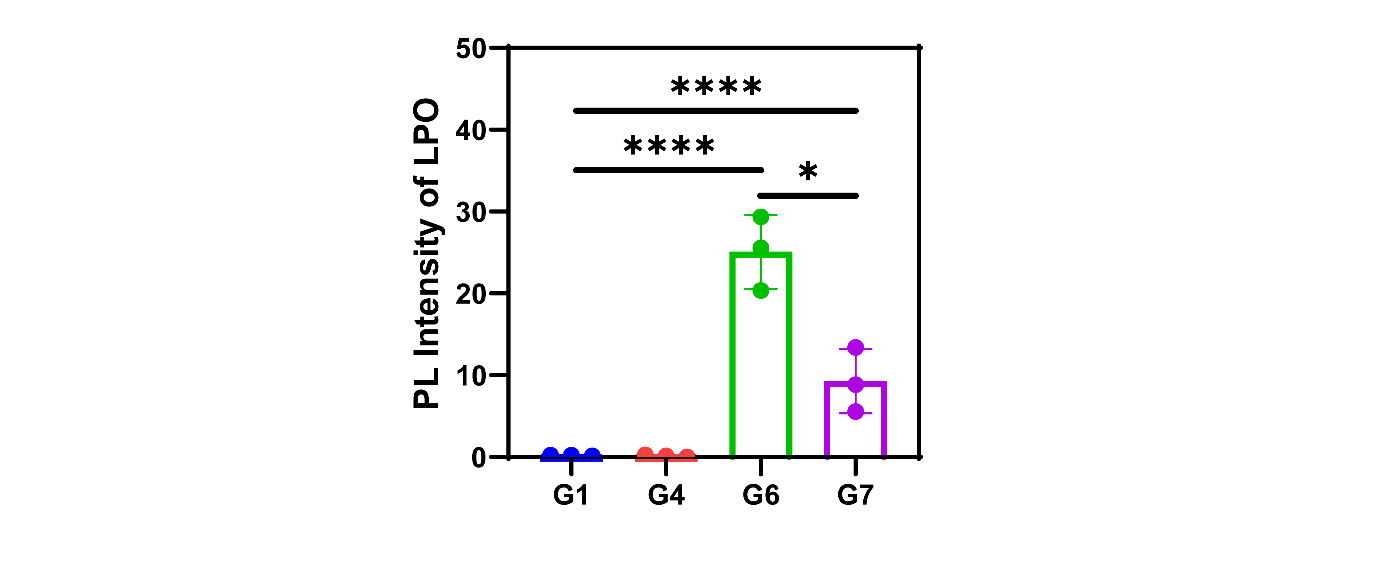


**Figure S22.** Quantitative data showing the mean fluorescence intensity (MFI) of LPO. According to the representative images in Figure 4A. Error bars: mean ± SD (n = 3). **p* < 0.05, ***p* < 0.01, ****p* < 0.001, *****p* < 0.0001.


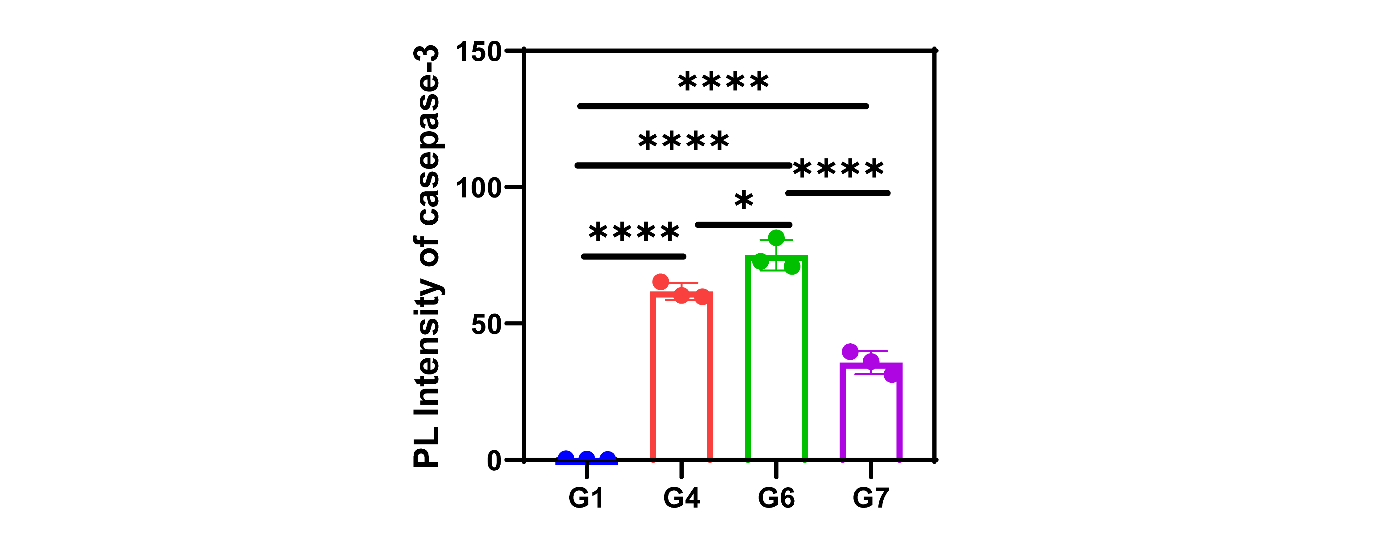


**Figure S23.** Quantitative data showing the mean fluorescence intensity (MFI) of casepase-3. According to the representative images in Figure 4A. Error bars: mean ± SD (n = 3). **p* < 0.05, ***p* < 0.01, ****p* < 0.001, *****p* < 0.0001.


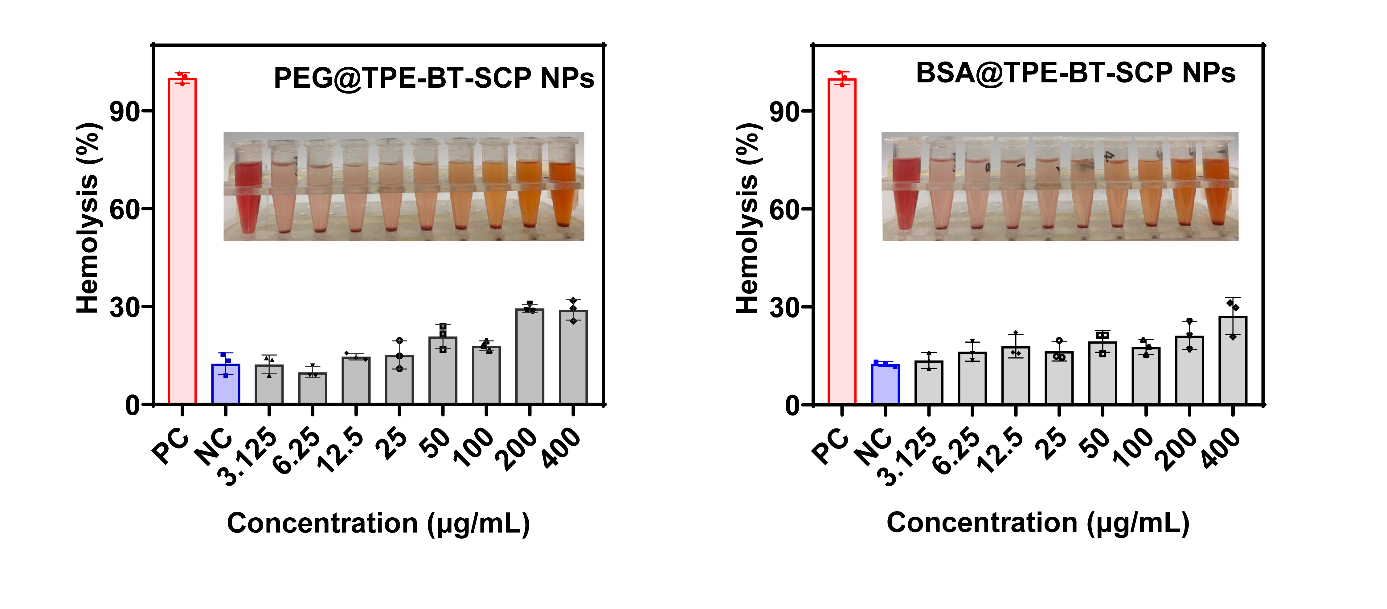


**Figure S24.** Hemolysis rate of different concentrations of PEG@TPE-BT-SCP NPs and BSA@TPE-BT-SCP NPs. The data are presented as the mean ± SD, n = 3.


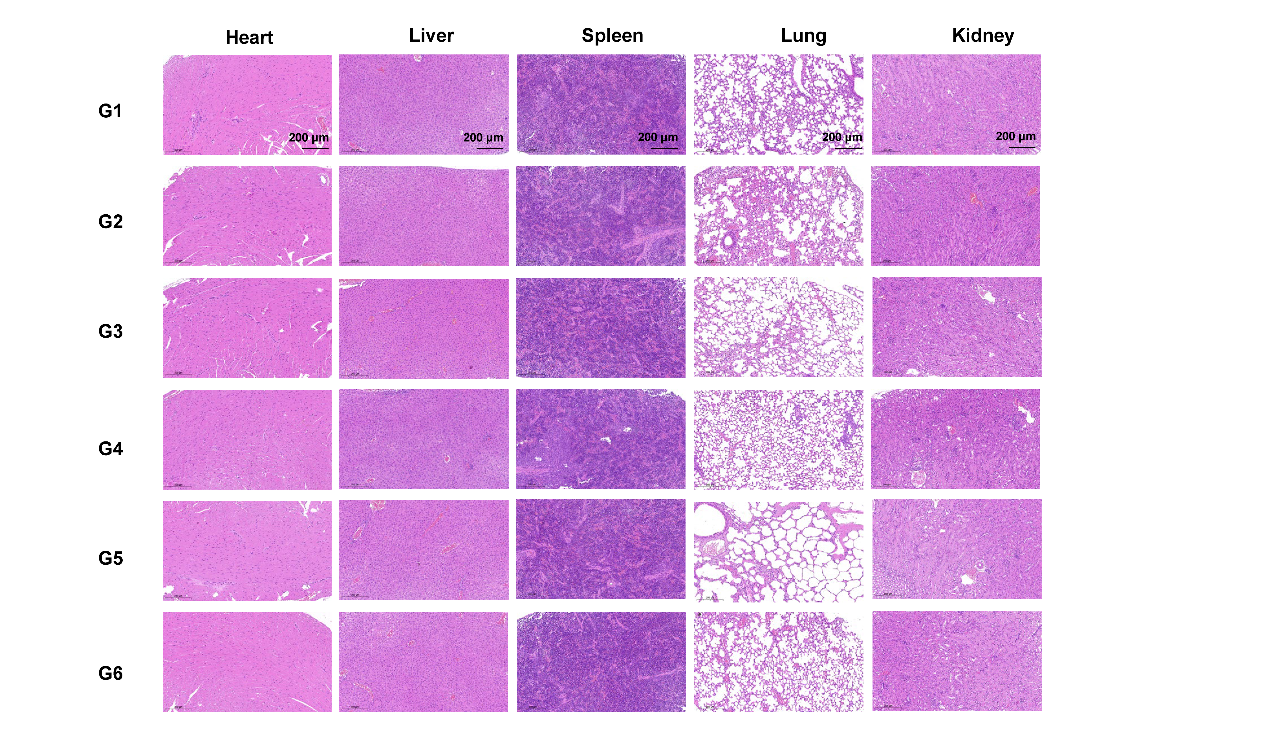


**Figure S25.** Typical images of H&E-stained main organs slices in different groups.


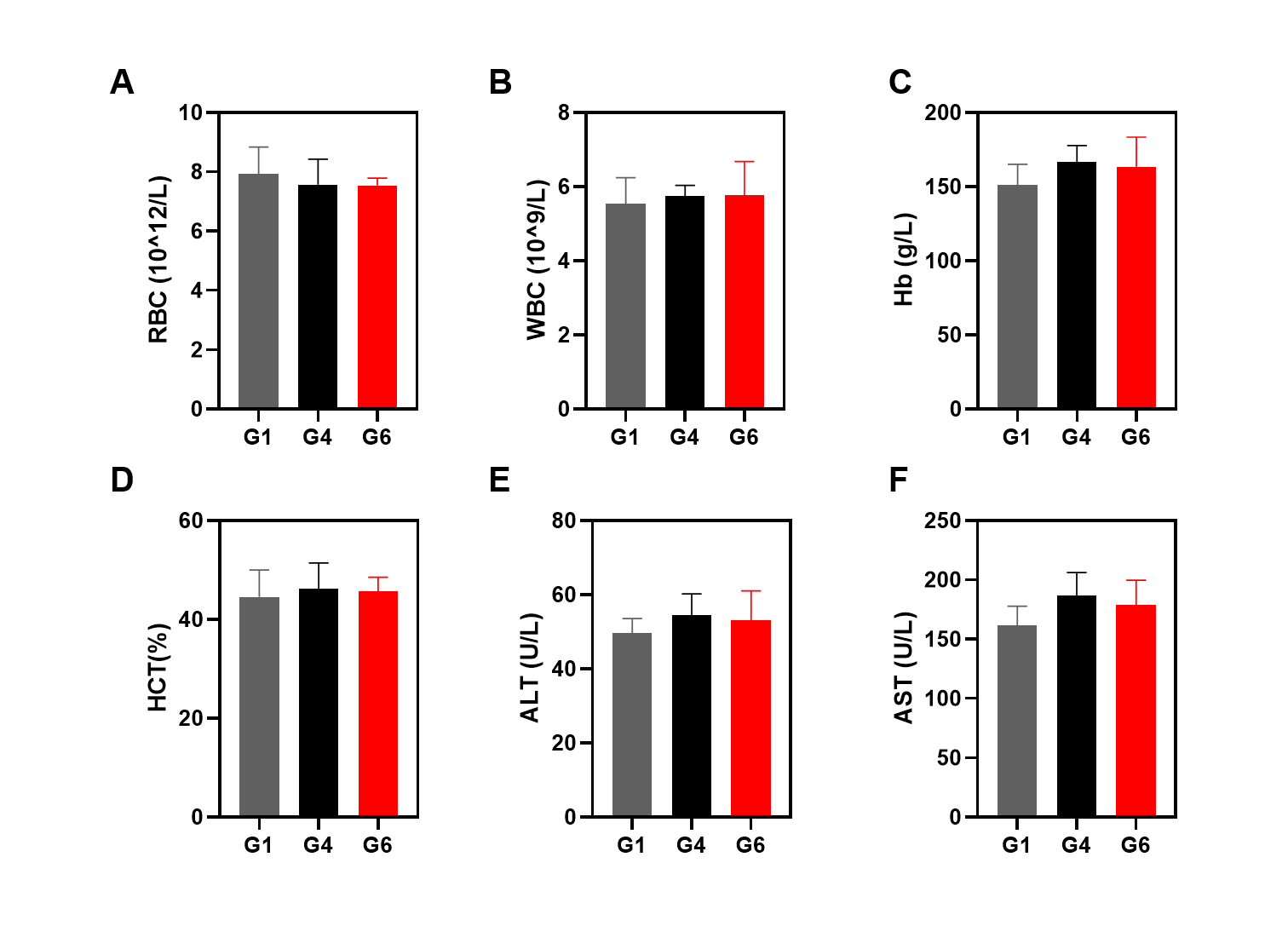


**Figure S26.** (A-D) Blood chemistry test parameters in terms of haem regulation and red or white blood cell counts of the mice from different groups. (E-F) Blood chemistry test parameters in terms of liver function of the mice from different groups.


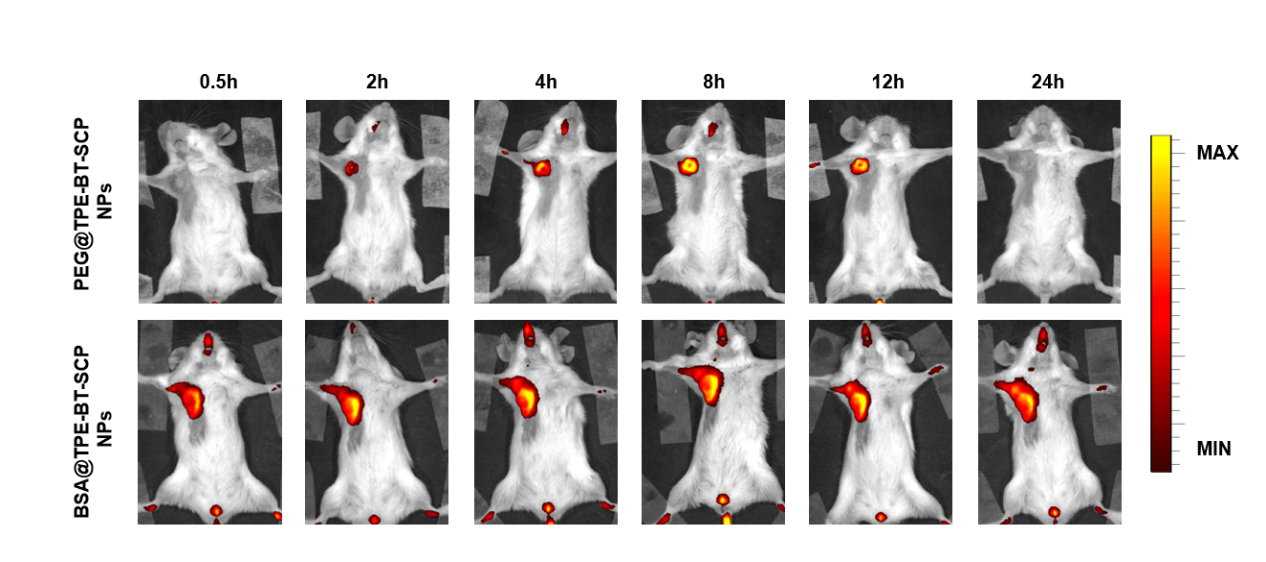


**Figure S27.** IVIS images of mouse at various time points post intravenous injection of PEG@TPE-BT-SCP NPs and BSA@TPE-BT-SCP NPs.


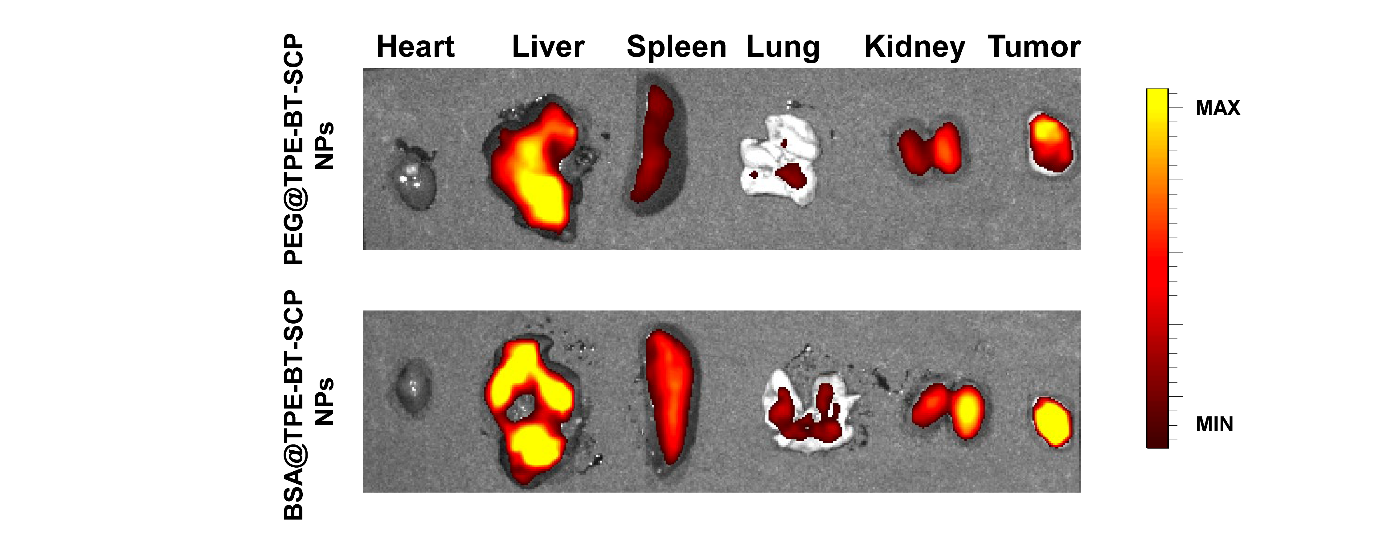


**Figure S28.** IVIS images of *ex vivo* organs collected at 8 h post intravenous injection.


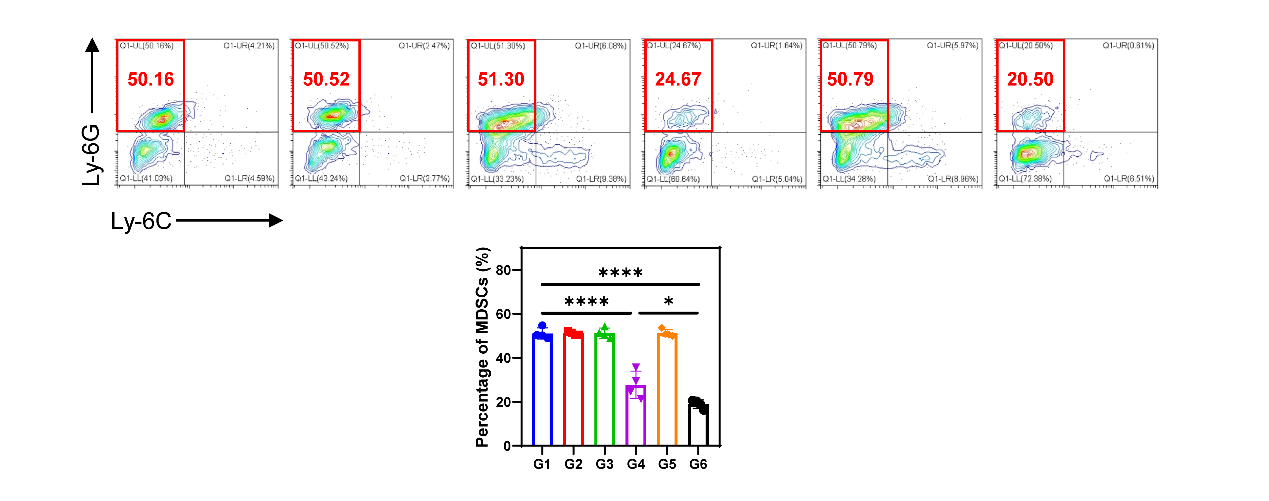


**Figure S29.** Representative flow cytometry plots of the proportions and quantitative analyses of MDSC cells in the tumors collected from 4T1 tumor-bearing mice in various groups.


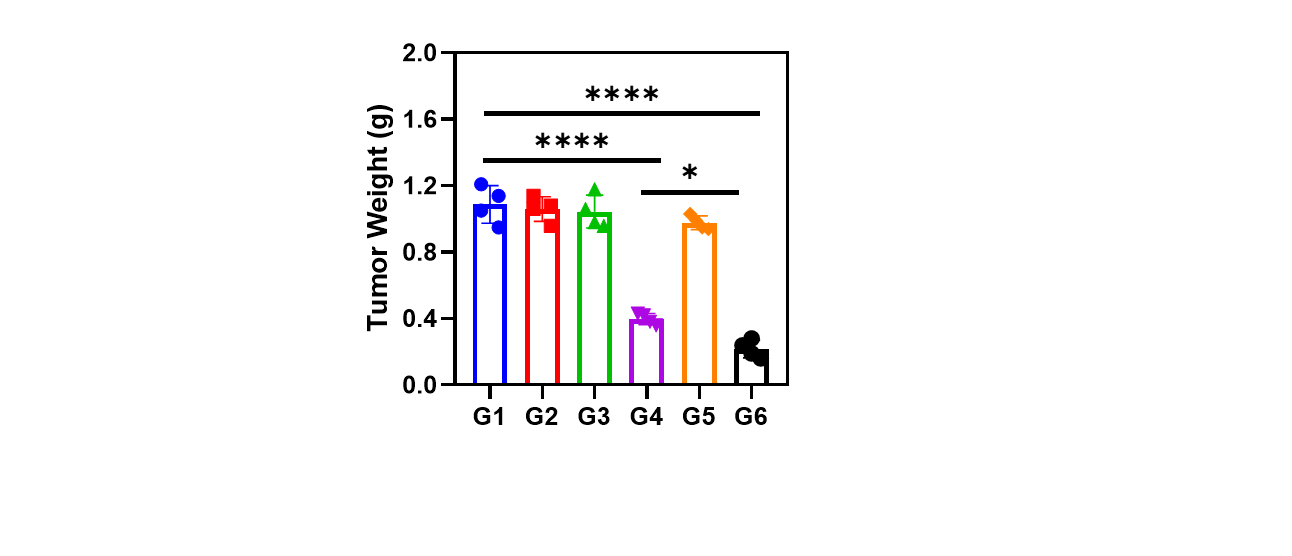


**Figure S30.** Weights of tumor of MM humanized mice in different groups.

**
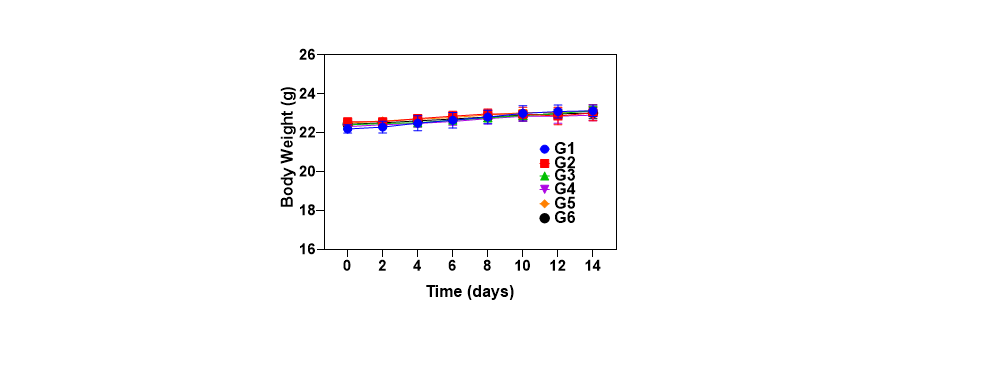
**

**Figure S31.** Body weight changes of MM humanized mice in different groups.


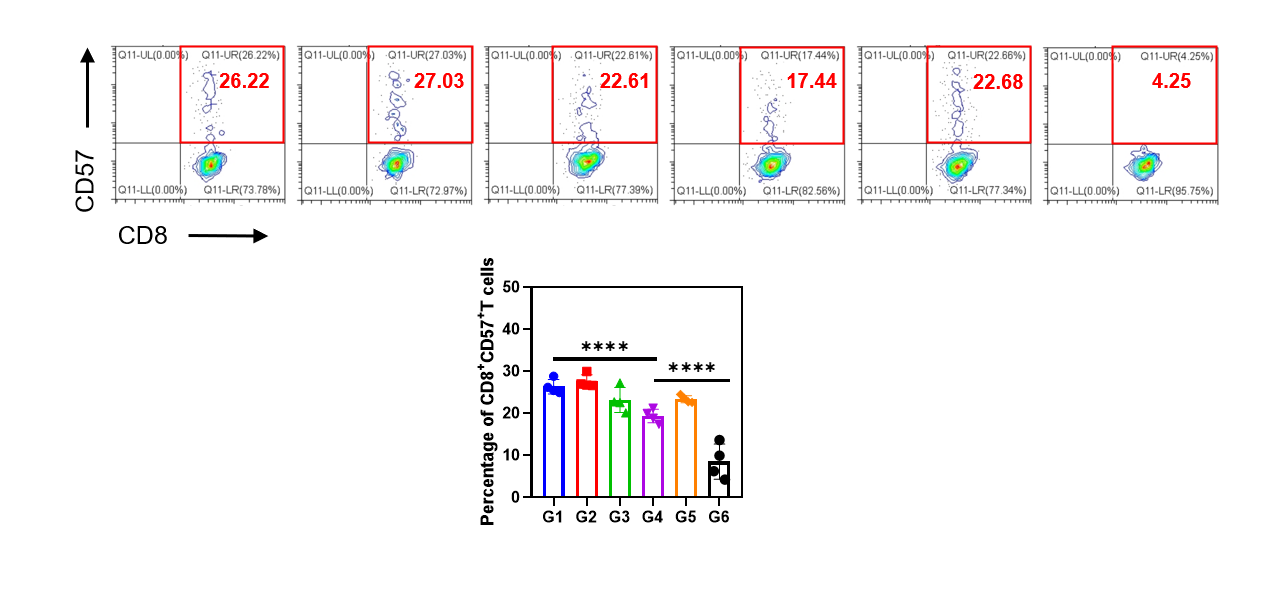


**Figure S32.** Representative flow cytometry plots of the proportions and quantitative analyses of senescent T cells in the tumors collected from MM humanized mice in various groups.
